# Transcriptomic responses of a simplified soil microcosm to a plant pathogen and its biocontrol agent reveal a complex reaction to harsh habitat

**DOI:** 10.1186/s12864-016-3174-4

**Published:** 2016-10-27

**Authors:** Michele Perazzolli, Noemí Herrero, Lieven Sterck, Luisa Lenzi, Alberto Pellegrini, Gerardo Puopolo, Yves Van de Peer, Ilaria Pertot

**Affiliations:** 1 Department of Sustainable Ecosystems and Bioresources, Research and Innovation Centre, Fondazione Edmund Mach, Via E. Mach 1, 38010 S Michele all’Adige, Italy; 2 Department of Plant Systems Biology, VIB, 9052 Ghent, Belgium; 3 Department of Plant Biotechnology and Bioinformatics, Ghent University, 9052 Ghent, Belgium; 4 Bioinformatics Institute Ghent, Ghent University, 9000 Ghent, Belgium; 5 Department of Genetics, Genomics Research Institute, University of Pretoria, Hatfield Campus, 0028 Pretoria, South Africa; 6 Present Address: Institute of Entomology, Biology Centre-The Czech Academy of Sciences, Branišovská 31/1160, České Budějovice, 37005 Czech Republic

**Keywords:** Soil microbial community, Soil transcriptome, Biological control, Plant pathogen, Microbial interaction, RNA-Seq, Transcriptomics, Gene expression

## Abstract

**Background:**

Soil microorganisms are key determinants of soil fertility and plant health. Soil phytopathogenic fungi are one of the most important causes of crop losses worldwide. Microbial biocontrol agents have been extensively studied as alternatives for controlling phytopathogenic soil microorganisms, but molecular interactions between them have mainly been characterised in dual cultures, without taking into account the soil microbial community. We used an RNA sequencing approach to elucidate the molecular interplay of a soil microbial community in response to a plant pathogen and its biocontrol agent, in order to examine the molecular patterns activated by the microorganisms.

**Results:**

A simplified soil microcosm containing 11 soil microorganisms was incubated with a plant root pathogen (*Armillaria mellea*) and its biocontrol agent (*Trichoderma atroviride*) for 24 h under controlled conditions. More than 46 million paired-end reads were obtained for each replicate and 28,309 differentially expressed genes were identified in total. Pathway analysis revealed complex adaptations of soil microorganisms to the harsh conditions of the soil matrix and to reciprocal microbial competition/cooperation relationships. Both the phytopathogen and its biocontrol agent were specifically recognised by the simplified soil microcosm: defence reaction mechanisms and neutral adaptation processes were activated in response to competitive (*T. atroviride*) or non-competitive (*A. mellea*) microorganisms, respectively. Moreover, activation of resistance mechanisms dominated in the simplified soil microcosm in the presence of both *A. mellea* and *T. atroviride*. Biocontrol processes of *T. atroviride* were already activated during incubation in the simplified soil microcosm, possibly to occupy niches in a competitive ecosystem, and they were not further enhanced by the introduction of *A. mellea*.

**Conclusions:**

This work represents an additional step towards understanding molecular interactions between plant pathogens and biocontrol agents within a soil ecosystem. Global transcriptional analysis of the simplified soil microcosm revealed complex metabolic adaptation in the soil environment and specific responses to antagonistic or neutral intruders.

**Electronic supplementary material:**

The online version of this article (doi:10.1186/s12864-016-3174-4) contains supplementary material, which is available to authorized users.

## Background

The soil microbial community is probably the most important component of the soil biota and it is responsible for a wide range of ecologically significant functions [1]. Soil microorganisms are key determinants of biological soil quality, supporting soil fertility and indirectly plant health [1]. Soil bacteria and fungi have undergone evolution and niche differentiation, interacting in different ways with various outcomes in soil ecosystems [2]. Microbe-microbe interactions have an important impact on microbial fitness in the soil [3] and they can positively or negatively affect other participants, including organisms of higher taxa [4]. These interactions can be synergistic, neutral or antagonistic, and can affect the growth, metabolism and differentiation of members of the microbial community [5]. For example, direct and indirect competition, like antagonism and nutrient consumption, have an adverse effect on interacting members of the population [5]. Plant pathogens have to compete with members of the rhizosphere microbiota for available nutrients and microsites in order to infect root tissue, and they are significantly restricted in growth by the antagonistic activities of biocontrol microorganisms in suppressive soils [6]. However, microbes have evolved strategies not only to fight each other, but in some cases relationships to adapt or support each other, increasing the overall fitness of the community [5]. Despite the widespread occurrence of interspecific microbial interactions in nature and their crucial relevance, there is a lack of understanding about how each species perceives and responds to interactions with other microbes within a complex community [7].

Molecular interplay between soil microorganisms has mainly been studied in dual cultures of plant pathogens and biocontrol agents [2, 7–10]. Soil phytopathogenic fungi cause severe diseases in several crops, and *Armillaria* spp. are among the most serious plant root pathogens, and cause significant economic losses to perennials, such as fruit crops, timber and ornamental trees in boreal, temperate and tropical regions of the world [11]. In particular, one of the most aggressive species (*Armillaria mellea*) colonises living woody roots, causing damping off and root rot [12]. Infected plants show a decline in vigour and production and generally die within a few years of infection [13]. *Armillaria mellea* can persist saprotrophically in roots after the death of the host and it can also spread below ground through specialised and robust structures known as rhizomorphs [12]. Fumigants and fungicides cannot completely eradicate the *Armillaria* spp. inoculum in soil, and post-infection control is commonly unsuccessful, as in the case of several soil phytopathogens [14]. Therefore, biocontrol microorganisms have been increasingly screened for antagonism against this soil phytopathogenic fungus. *Trichoderma* spp. are one of the most widely applied biocontrol fungi and the *Trichoderma atroviride* SC1 strain has been identified as an effective biocontrol agent of *A. mellea* [15]. The biocontrol properties of this strain have been studied in vitro, demonstrating that its mechanism of action relies mainly on hyperparasitization of *A. mellea* hyphae [16]. The biocontrol properties of *Trichoderma* spp. have been extensively studied using several approaches, including transcriptomics [17]. Most transcriptomic studies of microbial biocontrol mechanisms have focused on molecular responses of the biocontrol agent and the target plant pathogen in dual cultures [2, 7, 8, 10, 18]. However, microbial properties in complex consortia often differ from those of their single components [19]. In particular, biocontrol agents should compete with indigenous communities to occupy niches and show appropriate biocontrol properties against the target pathogens [20]. Introduction of *T. atroviride* changes the microbial structure of the soil microflora during the first two weeks following inoculation [21], suggesting strong interaction between this biocontrol strain and indigenous microbial communities. The objective of this study was to clarify the transcriptomic response of a simplified soil microcosm composed of 11 soil microorganisms to the introduction of a plant pathogen (*A. mellea*) and its biocontrol agent (*T. atroviride*) in a soil matrix. Greater insight into these mechanisms can help to understand the dynamics underlying interactions among soil microorganisms following the application of biocontrol agents against soil phytopathogenic fungi, and the impact of biocontrol agents on indigenous non-target microorganisms.

## Methods

### Growth conditions for soil microorganisms

*Azorhizobium caulinodans* ORS 571 (DSM 5975, [22])*, Bacillus subtilis* subsp. *subtilis* 168 (ATCC 23857, [23]), *Cupriavidus metallidurans* CH34 (DSM 2839, [22]) and *Pseudomonas protegens* Pf-5 (ATCC BAA477, [23]) were grown in 20 ml of Luria-Bertani broth (LB; Oxoid) at 25 °C overnight, with orbital shaking at 250 rpm. To prepare the inoculum suspension of each bacterial strain, 15 ml of the culture grown overnight were centrifuged at 5000 × g for 10 min at room temperature. The supernatant was discarded and the bacterial cells were washed three times by suspending in 20 ml of sterile isotonic solution (0.9 % NaCl) and centrifuging at 5000 × g for 10 min. The concentration of each bacterial suspension was assessed by measuring the optical density (OD) at 600 nm with a spectrophotometer (Ultrospec 3100, GE Healthcare Life Sciences) and adjusted to 5 × 10^8^ cells ml^−1^.

*Debaryomyces hansenii* CBS 767 [24], *Pichia stipitis* CBS 6054 [24], *Schizosaccharomyces pombe* 972 h (DSM 70576, [22]), *Saccharomyces cerevisiae* S288c (ATCC 204508, [23]) and *T. atroviride* SC1 (available in our strain collection and in the commercial product Vintec, Bi-PA, Londerzeel, Belgium) were grown on malt extract agar (MEA; Oxoid) at 25 °C. *Aspergillus niger* CBS 513.88 [24]*, Fusarium oxysporum* f. sp. *lycopersici* 4287 (CBS 123668, [24]) and *Penicillium chrysogenum* Wisconsin 54–1255 (DSM 1075, [22]), were grown on potato dextrose agar (PDA; Oxoid) at 25 °C. To prepare the inoculum suspension of filamentous fungi and yeasts, conidia and cells were collected from 21-day-old cultures by washing each plate with 3 ml of sterile isotonic solution, using a glass rod under sterile conditions. The suspension of each strain was filtered with sterile cloth, and cells were washed three times by suspending in 6 ml of sterile isotonic solution and centrifuging at 5000 × g for 10 min. The concentration of each conidia and cell suspension was adjusted to 5 × 10^8^ cells ml^−1^ by counting with a hemocytometer under a light microscope. For the inoculum suspension of *A. mellea* M6132 (kindly provided by Dr. Simone Prospero, Swiss Federal Research Institute, Birmensdorf), 1 g of mycelium was collected from 21-day-old culture grown on MEA at 25 °C and suspended in 4 ml of sterile isotonic solution. Sterile steel beads were added and the *A. mellea* mycelium was ground in a mixer-mill disruptor (MM 400, Retsch) at 25 Hz for 3 min. The ground mycelium was washed three times by suspending in 4 ml of sterile isotonic solution and centrifuging at 5000 × g for 10 min, and it was then suspended in 2 ml of sterile isotonic solution.

The viability of counted cells in each inoculum suspension was validated through a dilution plating method (Additional file 1). Basically, 0.1 ml of each inoculum suspension was subjected to 10-fold serial dilution, 0.2 ml of each dilution was plated in triplicate on plates containing the appropriate media for each strain, bacterial and fungal colony forming units (CFUs) were then assessed after incubation at 25 °C for 24 and 48 h respectively.

### Set-up of the simplified soil microcosm and sample collection

The soil matrix was obtained as described by Ellis [25], by mixing 35 g of sand (Sigma-Aldrich), 10 g of kaolinite (Sigma-Aldrich), 5 of bentonite (Sigma-Aldrich) and 0.1 g of CaCO_3_ (Sigma-Aldrich) in a 200 mL glass vessel for plant tissue culture (Sigma-Aldrich). The soil matrix was sterilised at 121 °C for 30 min, dried in an oven at 80 °C for 24 h and cooled down to room temperature. Subsequently, 1 g of humic acid (Sigma-Aldrich) was added under sterile conditions and the soil matrix was mixed by vigorous shaking.

To prepare the simplified soil microcosm, the soil matrix was inoculated with a suspension obtained by mixing 1 ml of the suspension of each microbial strain to a final volume of 15 ml in sterile isotonic solution, and it was incubated at 25 °C with orbital shaking at 150 rpm. Five conditions were analysed in triplicate: the simplified soil microcosm incubated for 24 h either without exogenous fungi (SSM), with the biocontrol agent *T. atroviride* (SSM+T), with the plant pathogen *A. mellea* (SSM+A) or with both (SSM+T+A), and the simplified soil microcosm collected at the beginning of the experiment just after inoculation of the 11 soil microorganisms (SSM_0_). For each condition, 20 aliquots of 2 g of soil were collected and placed into 15 ml sterile tubes, immediately frozen in liquid N_2_ and stored at −80 °C. In an preliminary experiment the viability of each microbial strain after 24 h of incubation in the soil matrix was validated (Additional file 1). Specifically, each microbial strain was incubated in the soil matrix at 25 °C, after 24 h the CFUs were assessed by a dilution plating method on plates containing the above mentioned media and no significant changes (*t*-test, *P* > 0.05) on microbial CFUs were found for each soil microorganism before and after incubation in the soil matrix.

### RNA isolation and mRNA enrichment

Total RNA was extracted from 2 g of soil using the RNA PowerSoil Total RNA Isolation Kit (MoBio Laboratories) with slight modifications. Briefly, beads were directly added to the frozen soil sample, and the bead solution was added simultaneously with Solution SR1. After vortexing, the sample was centrifuged at 2500 × g for 20 min at room temperature and the following steps of the extraction protocol were carried out, according to the manufacturer’s instructions. Total RNA was quantified using a Nanodrop 8000 (Thermo Fisher Scientific) and its quality was checked using a Bioanalyzer 2100 (Agilent Technologies). In a preliminary experiment, each soil microorganism was separately inoculated in the soil matrix and the equivalent quality and yield of total RNA were assessed 24 h after incubation at 25 °C.

For each of the five conditions, twelve independent extractions of total RNA were obtained from the simplified soil microcosm, and three replicates deriving from the pool of four RNA extractions were used in further analysis. Total RNA was purified and concentrated using the RNeasy Plus Micro kit (Qiagen), which allowed depletion of genomic DNA, and 3 μg total RNA was subjected to mRNA enrichment. The RiboMinus Eukaryote Kit for RNA-Seq (Invitrogen, Life Technologies) was used, and probes of the Ribominus Transcriptome Isolation Kit (Yeast and Bacteria) (Invitrogen, Life Technologies) were added in the hybridization step, in order to simultaneously remove the ribosomal RNA of fungi, bacteria and yeasts. Purified mRNA was quantified using a Nanodrop 8000 (Thermo Fisher Scientific) and its quality was checked using a Bioanalyzer 2100 (Agilent Technologies) to verify the absence of ribosomal RNA.

### Library construction and Illumina sequencing

Three replicates for each condition were subjected to RNA-Seq library construction, using the TruSeq SBS v3 protocol (Illumina), and paired-end reads of 100 nucleotides were obtained using an Illumina HiSeq 2000 at Fasteris (Plan-les-Ouates, Switzerland). Briefly, 200 ng of the purified mRNA was fragmented using Zn-catalysed hydrolysis and converted into double-stranded cDNA with random priming. Following end repair, indexed adapters were ligated and cDNA fragments of 200 ± 50 bp were purified. Purified cDNA was amplified by PCR and validated by Illumina sequencing, after which mRNA-Seq libraries were multiplexed (four libraries per lane) and sequenced according to the manufacturer’s instructions.

### Mapping of sequenced reads and assessment of gene expression

Quality trimming and sequence filtering were carried out using Trimmomatic (version 0.32) [26] to remove sequencing adapters, low-quality bases at the end of the reads (Phred quality score lower than 15 in a sliding window of 4 bases) and short reads (shorter than 50 bases or with an average Phred quality score below 25). The FastQC (version 0.10.1) was used for sequence quality control [27].

The reference genomes of the 13 microorganisms were downloaded from public databases (Table 1), unique identifiers of contigs and genes were generated by adding a two letter code for each microorganism and the microcosm genome was built by merging the genomic data. Filtered read pairs were mapped to the microcosm genome using TopHat2 (version 2.0.10) [28] with the default settings, except for the inner distance between mate pairs of 200 ± 50, maximum intron length of 4000, and minimum intron length of 10 [29]. The results of the alignments were analysed with Samtools (version 0.1.19) [30] and visualised using the Integrative Genomics Viewer [31]. Unambiguously mapped read pairs were used to improve the specificity of gene expression estimation, and read pairs mapped to gene regions were counted using HTSeq [32]. As for transcriptomic studies of microbial consortia [33, 34], global normalisation of read mapping to the microcosm genome was used and gene expression levels were calculated as fragments per kilobase of each gene per million of unambiguously mapped read pairs (FPKM; [35]) of the microcosm genome. Using this approach, changes in gene expression levels are related to transcriptional regulation and the biomass abundance of each microorganism, compared with other members of the microcosm [36].

**Table 1:** Genomic features of the microorganisms used in the simplified soil microcosm

Microbial strain^a^	Type^b^	Main biological characteristics of microbial species	Genome version	Reference and database^c^	Genome features^d^	
					Length	Genes
*Azorhizobium caulinodans* ORS 571	B	Gram-negative, nitrogen fixing bacterium both free-living in soil and in symbiotic interaction with plants	GCA_000010525.1	[54, 86]	5.4	4781
*Bacillus subtilis* subsp. *subtilis* 168	B	Gram-positive, common in soil and the rhizosphere	GCA_000009045.1	[55, 87]	4.2	4371
*Cupriavidus metallidurans* CH34	B	Gram-negative, also found in soil, adaptable to heavy metal stress	GCA_000196015.1	[56, 88]	6.9	6829
*Pseudomonas protegens* Pf-5	B	Gram-negative, common in soil, biocontrol agent	GCA_000012265.1	[57, 89]	7.1	6212
*Debaryomyces hansenii* CBS 767	Y	Salt-tolerant multilateral budding yeast of water and soil	v1.0	[58, 90]	12.1	6272
*Pichia stipitis* CBS 6054	Y	Budding yeast in soil and beetle gut	v2.0	[60, 91]	15.4	5807
*Saccharomyces cerevisiae* S288c	Y	Ubiquitous budding yeast, also found in soil	R64-1-1	[44, 45]	12.2	7126
*Schizosaccharomyces pombe* 972 h	Y	Fission yeast, isolated from soil	ASM294v2	[61, 92]	12.6	7018
*Aspergillus niger* CBS 513.88	FF	Ubiquitous in soil, saprophyte and pathogen of plants	CADRE	[62, 93]	33.9	14,445
*Fusarium oxysporum* f. sp. *lycopersici* 4287	FF	Ubiquitous in soil, phytopathogen	FO2	[64, 94]	60.0	18,066
*Penicillium chrysogenum* Wisconsin 54-1255	FF	Ubiquitous, also found in soil, equipped with antifungal properties	v1.0	[63, 95]	32.2	13,671
*Trichoderma atroviride* IMI 206040^e^	BA	Ubiquitous, also found in soil, biocontrol agent	TRIAT v2.0	[65, 96]	36.1	11,863
*Armillaria mellea* DSM 3731^f^	PP	Soil inhabitant and polyphagous plant root pathogen	v1.0	[66, 97]	84.2	14,473
Microcosm genome^g^					322.2	120,934

^a^The simplified soil microcosm was obtained through inoculation of four bacteria (B), four yeasts (Y) and three filamentous fungi (FF) with sequenced genomes into a soil matrix. The plant pathogen (PP) and its biocontrol agent (BA) were also introduced

^b^Soil microorganisms are classified as: bacterium (B), yeast (Y), filamentous fungus (FF), biocontrol agent (BA) and plant pathogen (P)

^c^References and databases of the downloaded genome versions

^d^Genome length (Mbp) and number of genes in the reference genome

^e^The reference genome of *T. atroviride* IMI 206040 was used to align read pairs deriving from *T. atroviride* SC1

^f^The reference genome of *A. mellea* DSM 3731 was used to align read pairs deriving from *A. mellea* M6132

^g^The microcosm genome was obtained by merging the data of 13 soil microorganisms and was used as a reference for RNA-Seq alignment

### Identification of differentially expressed genes

Differentially expressed genes (DEGs) were identified with the DESeq2 package [37]. Briefly, counts of unambiguously mapped reads were normalised for sequencing depth following negative-binomial distribution and an empirical Bayes shrinkage approach was used for estimation of data dispersion and fold changes [37]. A false discovery rate (FDR) of Benjamini-Hochberg multiple tests lower than 5 % (*p* < 0.05) with a minimum Log2 fold change of one and a minimum expression level of 0.6 FPKM (corresponding to a coverage of 1× of the gene sequence in our samples) in at least one condition were imposed to identify DEGs through pairwise comparison. Four pairwise comparisons of the simplified soil microcosm were analysed: SSM vs. SSM_0_, SSM+A vs. SSM_0_, SSM+T vs. SSM_0_, and SSM+T+A vs. SSM_0_. DEGs were then grouped according to their expression profiles and clusters were visualised using MultiExperiment Viewer software (version 4.9) [38]. Two additional pairwise comparisons (SSM+A vs. SSM+T+A and SSM+T vs. SSM+T+A) were considered to identify the DEGs of *A. mellea* and *T. atroviride*. Fold changes of FPKM values were calculated, with the minimum expression value of 0.002 FPKM imposed on non-expressed genes.

### Functional annotation of differentially expressed genes

DEGs were annotated using the ARGOT2 function prediction tool [39], and grouped into 17 functional categories of Gene Ontology (GO) biological process terms [40], based on the BlastX results against the UniProtKB database [41]. Genes not associated with any biological process category were assigned to the GO root (GO:0008152, namely biological process). Gene descriptions were obtained from the annotation files available in public databases for the 13 soil microorganisms (Table 1). To improve gene descriptions, sequences of DEGs were aligned using a BlastX search against the Swiss Prot database [41], and the best hits (E-value lower than 1E^−5^) were manually checked.

GO terms significantly overrepresented in the DEGs of the simplified soil microcosm were identified using the Biological Networks Gene Ontology (BiNGO) tool [42], and biological networks were visualised with Cytoscape version 3.2.1 [43]. Basically, sequences of DEGs were aligned against the protein database of *S. cerevisiae* S288c [44, 45] using a BlastX search and homologous *S. cerevisiae* proteins were identified (E-value lower than 1E^−5^). Redundancies were removed and significantly overrepresented (*P* < 0.05) GO biological process terms were identified for the up- and down-regulated genes of each cluster in comparison with the whole *S. cerevisiae* annotation as a reference set.

DEGs were also assigned to KEGG (Kyoto Encyclopedia of Genes and Genomes) ortholog groups, redundancies were removed and they were mapped to KEGG pathways using the KEGG Automatic Annotation Server (KAAS) [46, 47] with the BBH method and default gene data set [48, 49].

### Gene expression analysis by quantitative real-time RT-PCR

First-strand cDNA was synthesized from 600 ng of purified RNA using SuperScript VILO with random hexamers (Invitrogen, Life Technologies). cDNA was tested for contamination by genomic DNA through amplification of a translocon alpha subunit Sec61 of *S. pombe* with intron-spanning primers (Additional file 2). PCR reaction was carried out with a T-Professional Thermocycler (Biometra) using the DreamTaq DNA Polymerase (Fermentas, Life Technologies), and amplification of the expected cDNA fragment of Sec61 was verified with gel electrophoresis.

Quantitative real-time PCR (RT-qPCR) was carried out on ten times-diluted cDNA with Platinum SYBR Green qPCR SuperMix-UDG (Invitrogen, Life Technologies) and specific primers (Additional file 2), using a LightCycler 480 instrument (Roche, Branford, CT, USA). Reactions were set with two initial steps at 55 °C for 10 min and 95 °C for 2 min, followed by 50 cycles at 95 °C for 15 s and 60 °C for 1 min. Each sample was examined in three technical replicates and dissociation curves were analysed to verify the specificity of each amplification reaction. To validate the amplification of the target genes, real-time PCR products were treated with Exosap-IT (Usb Corporation) and sequenced on both strands using an AB3730xl instrument (Applied Biosystems) at the Sequencing Platform facility (Fondazione Edmund Mach). Sequences were examined using FinchTV Version 1.4.0 (Geospiza Inc.) and aligned with the expected target gene using T-Coffee Multiple Sequence Alignment [50]. The specificity of designed primers and amplified sequences was further validated with a BlastN search against all genes of the microcosm composed by 13 soil microorganisms, and specific alignments to each target gene were verified.

Cycle threshold Ct) values were extracted with LightCycler 480 SV1.5.0 software (Roche) using the second derivative calculation. For each gene, the reaction efficiency (Eff) was calculated with the LinRegPCR 11.1 software [51] and used to calculate relative quantities (RQ), according to the formula: RQ = Eff^(Ct–Ct′)^, where Ct is the threshold cycle and Ct’ is the average Ct of all the conditions analysed [52]. Four housekeeping genes were selected, as their expression was not significantly affected in the five conditions (Additional file 2), and their stability was validated using the ∆Ct method described by Silver et al. [53]. Normalised relative quantities (NRQ) were then calculated by dividing the RQ by a normalisation factor, based on the RQ value of the two most stable reference genes [52]: *Actin 1* of *S. pombe* and *Elongation factor 1α* of *F. oxysporum*, for global normalisation of the microcosm transcriptome.

## Results

### Experimental design and RNA-Seq analysis of the simplified soil microcosm

The simplified soil microcosm was based on a soil matrix simultaneously inoculated with 11 soil microorganisms whose genomes were already sequenced and available that were selected to mimic a community of bacteria (Gram-positive and negative), yeasts (fission and budding yeasts) and filamentous fungi commonly found in soil [44, 54–66], having beneficial (biocontrol agent or nitrogen fixing microorganisms), pathogenic, saprophytic or neutral properties for crops (Table 1). The plant pathogen (*A. mellea*) and its biocontrol agent (*T. atroviride*) were introduced into the simplified soil microcosm and samples were collected 24 h after incubation under controlled conditions, to analyse the early transcriptional response of the microbial community to these exogenous fungi. Five conditions were analysed in triplicate: the simplified soil microcosm incubated for 24 h either without exogenous fungi (SSM), with the biocontrol agent *T. atroviride* (SSM+T), with the plant pathogen *A. mellea* (SSM+A) or with both (SSM+T+A), and the simplified soil microcosm collected at the beginning of the experiment just after inoculation of the 11 soil microorganisms (SSM_0_). From 46 to 80 million paired-end reads of 100 nucleotides were obtained for each replicate and more than 84 % of them passed the quality control test (filtered read pairs; Additional file 3). More than 96 % of filtered read pairs were aligned (mapped read pairs) to the microcosm genome obtained by merging the genomes of the 11 soil microorganisms, the plant pathogen and the biocontrol agent. From 18 to 27 % of mapped read pairs were unambiguously aligned to unique locations (unique read pairs, Additional file 3) and reads mapping to multiple locations were not considered for gene expression analysis, as they possibly matched orthologous genes with high sequence similarity among the 13 soil microorganisms analysed.

The majority (more than 77 %) of unique read pairs were mapped to gene regions of the microcosm genome (Additional file 3) and the proportion of read pairs mapping to genes revealed the different transcriptional activity of soil microorganisms (Additional file 4A–D). Specifically, high (more than 10 %) and low (less than 0.1 %) proportions of read pairs were mapped to the *P. protegens* and *B. subtilis* genes respectively, in agreement with the high and low number of CFUs inoculated in the soil matrix (Additional file 1). However, high proportions of expressed genes (more than 76 %) were obtained for underrepresented transcriptomes (Additional file 4E, F and Additional file 5), such as those of *A. caulinodans* and *D. hansenii* (less than 2 % of unique pairs mapping to genes; Additional file 4D). Proportion of expressed genes of each soil microorganism was consistent among the five conditions, indicating that transcriptional activity and viability were not affected by incubation in the artificial soil in the presence or absence of *A. mellea* and *T. atroviride*. As expected, read pairs of *A. mellea* and *T. atroviride* were found either in the SSM+A or SSM+T and in the SSM+A+T condition, confirming the specificity of the alignment results (Additional file 4B, D and F).

### Identification and functional annotation of differentially expressed genes

**Fig. 1:**
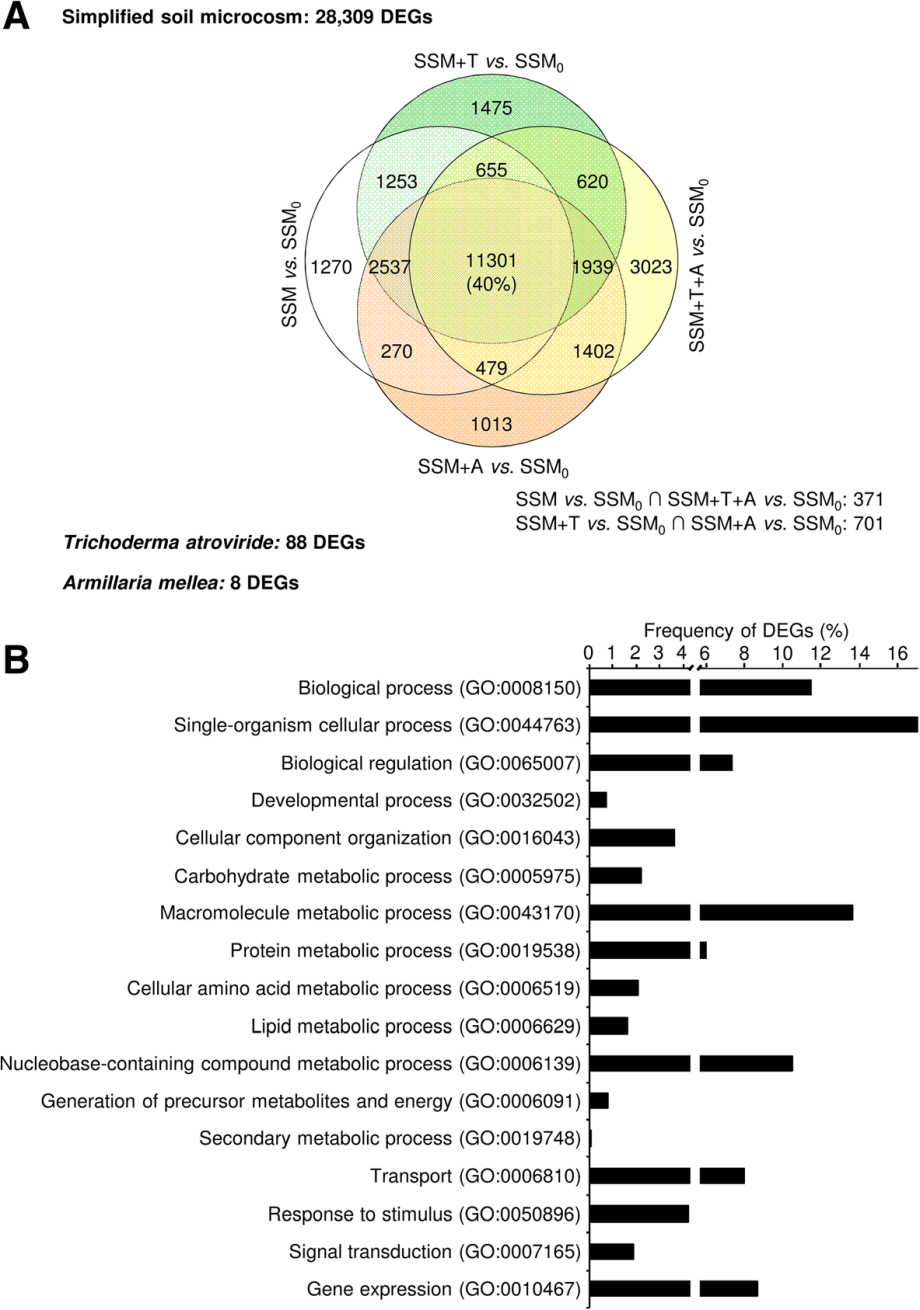
Differentially expressed genes of the simplified soil microcosm in response to *Trichoderma atroviride* and/or *Armillaria mellea* introduction. **a** Venn diagram summarising the distribution of 28,405 differentially expressed genes (DEGs) of the simplified soil microcosm identified by the DESeq2 package [37] in four pairwise comparisons of the simplified soil microcosm incubated for 24 h either without exogenous fungi (SSM), with the biocontrol agent *T. atroviride* (SSM+T), with the plant pathogen *A. mellea* (SSM+A) or with both (SSM+T+A), in comparison with the simplified soil microcosm collected at the beginning of the experiment (SSM_0_). Two additional pairwise comparisons (SSM+T+A vs. SSM+A and SSM+T+A vs. SSM+T) identified eight and 88 DEGs of *A. mellea* and *T. atroviride*, respectively. **b** Distribution of DEGs in the 17 selected Gene Ontology (GO) functional categories (GO identifiers are reported in *brackets*). Frequencies were calculated as a percentage of the total number of GO biological process terms obtained using the ARGOT2 function prediction tool [39]

Major transcriptional changes occurred during incubation of the soil microorganisms in the soil matrix, independently of *A. mellea* and *T. atroviride* introduction (Additional file 6). Pearson’s correlation coefficients of gene expression levels among the simplified soil microcosm collected at the beginning of the experiment (SSM_0_) and the four conditions at 24 h ranged from 0.85 to 0.87, and they were lower than those found among the four conditions at 24 h (ranging from 0.98 to 0.99). A total of 28,309 DEGs were found using DESeq statistical analysis, with a FDR of 5 %, a minimum fold-change of two and an expression level greater than 0.6 FPKM (Additional file 7). About 40 % of DEGs were expressed in all conditions, and a total of 3023 DEGs were specifically modulated by *A. mellea* and *T. atroviride* introduction (SSM+A+T; Fig. 1a). Moreover, eight and 88 DEGs of *A. mellea* and *T. atroviride* were identified in comparison of SSM+A and SSM+T vs. SSM+T+A respectively, and they represent transcriptional changes during pathogen-biocontrol interaction within the simplified soil microcosm. The proportion of DEGs ranged from 12 to 82 % of the expressed genes for each strain and it was not correlated with the proportion of expressed genes (Pearson’s correlation coefficient of 0.53, *P* = 0.06, Additional file 8). For example, *C. metallidurans* showed high transcriptional activity (91 % of expressed genes) and reduced transcriptional changes (38 % of DEGs), while *P. protegens* and *P. stipitis* were both highly transcriptionally active (more than 98 % of expressed genes) and responsive to the tested conditions (more than 64 % of DEGs).

Functional annotation of DEGs revealed regulation of microbial processes related to gene expression, nucleobase-containing compounds (DNA and RNA metabolism) and macromolecule metabolic processes during incubation in the soil matrix (Fig. 1b). Transcriptional regulations of the simplified soil microcosm included primary metabolism processes (carbohydrate, protein and lipid metabolic process), transport and cellular responses (response to stimulus and signal transduction), and they were stimulated by adaptation to the soil matrix environment and by *A. mellea* or *T. atroviride* introduction. However, a large fraction of DEGs (17 and 12 % respectively) were associated with poorly defined processes (single-organism cellular process) or were not associated with known functions (assigned to the Gene Ontology root, biological process).

### Expression profiles of the differentially expressed genes

**Fig. 2:**
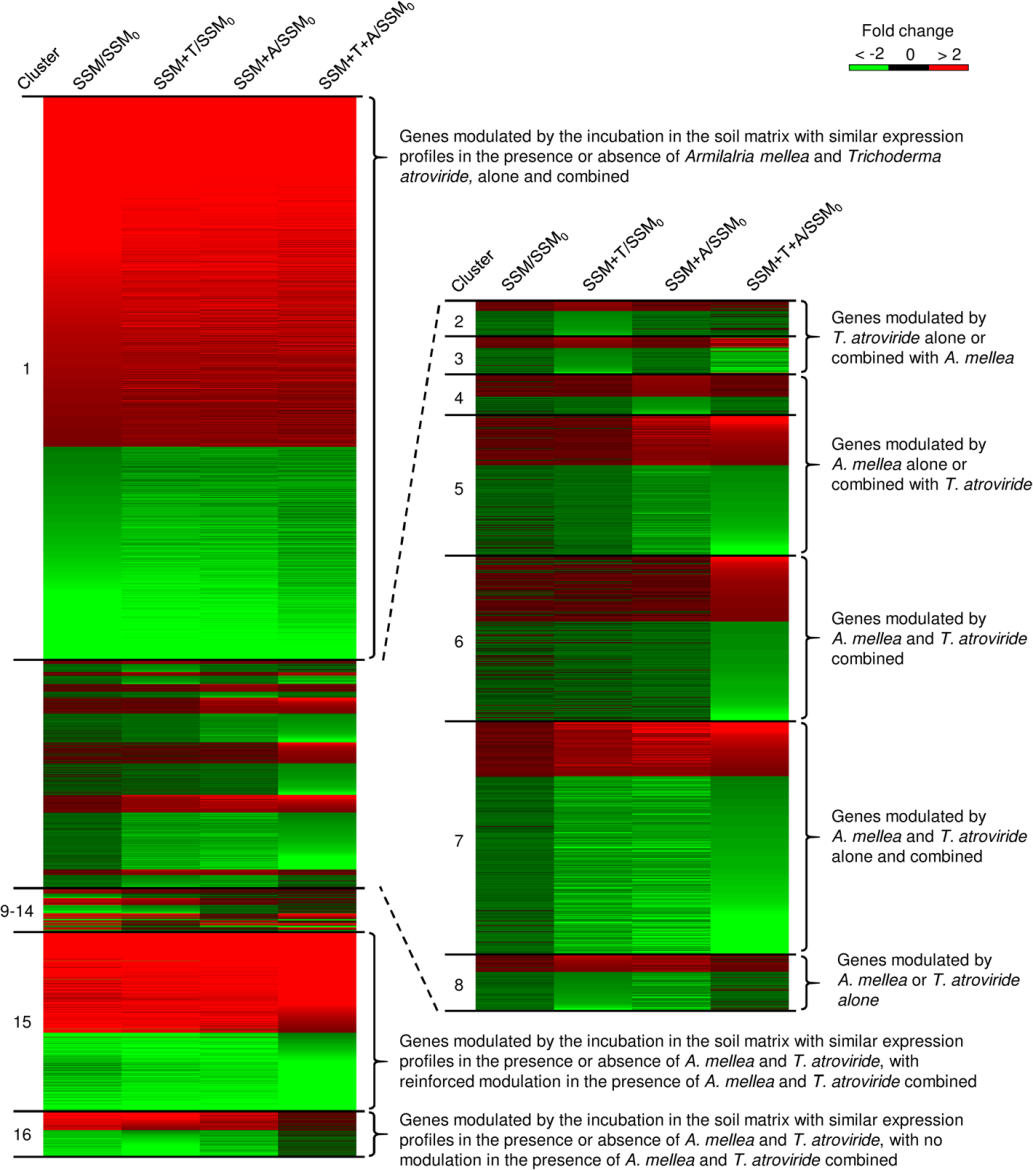
Clustering of differentially expressed genes (DEGs) of the simplified soil microcosm based on expression profiles. Heat map diagram of fold change values for the simplified soil microcosm incubated for 24 h either without exogenous fungi (SSM), with the biocontrol agent *Trichoderma atroviride* (SSM+T), with the plant pathogen *Armilalria mellea* (SSM+A) or with both (SSM+T+A), calculated as compared to the simplified soil microcosm collected at the beginning of the experiment (SSM_0_). Genes modulated by 24 h incubation in the soil matrix (SSM/SSM_0_ ≥ 2) were classified as: genes with similar expression profiles in the presence or absence of *A. mellea* and *T. atroviride* alone and combined (cluster 1), genes with similar expression profiles in the presence or absence of *A. mellea* and *T. atroviride* with reinforced modulation (cluster 15) or no modulation (cluster 16) in the presence of *A. mellea* and *T. atroviride* combined, and genes with specific profiles in the presence of *T. atroviride* and/or *A. mellea* (clusters 9–14). Genes not affected by incubation in the soil matrix (SSM/SSM_0_ < 2) were classified as: genes modulated by *T. atroviride* alone (cluster 2) or combined with *A. mellea* (cluster 3), and genes modulated by *A. mellea* alone (cluster 4) or combined with T. atroviride (cluster 5). Three clusters grouped genes modulated by the presence of *A. mellea* and *T. atroviride* combined (cluster 6), by the presence of *A. mellea* and *T. atroviride* alone and combined (cluster 7), and by the presence of *A. mellea* or *T. atroviride* alone (cluster 8). The heat map diagram was visualised using MultiExperiment Viewer [38] according to the colour scale legend shown

The DEGs of the simplified soil microcosm were grouped into 16 clusters based on the expression profiles (clusters 1–16; Fig. 2), and two additional clusters included the DEGs of *T. atroviride* (cluster 17) and *A. mellea* (cluster 18; Additional file 9). The majority (53 %) of DEGs of the simplified soil microcosm were modulated by incubation in the soil matrix, with similar expression profiles in the presence or absence of *A. mellea* and *T. atroviride*, alone and combined (cluster 1), and reflected adaptation processes to the soil matrix environment not influenced by the presence of the plant pathogen and the biocontrol agent. A large fraction (17 and 4 % respectively) of DEGs were modulated by incubation in the soil matrix, with similar expression profiles in the presence or absence of *A. mellea* and *T. atroviride*, with reinforced modulation (cluster 15) or no modulation (cluster 16) in the presence of *A. mellea* and *T. atroviride* combined. Of the DEGs of the simplified soil microcosm, 322 and 327 genes were modulated by *T. atroviride* alone (cluster 2) or combined with *A. mellea* (cluster 3), respectively. Moreover, 346 and 1202 genes were modulated by *A. mellea* alone (cluster 4) or combined with *T. atroviride* (cluster 5), respectively. Cluster 6 comprised 1407 genes whose expression was affected by the presence of *A. mellea* and *T. atroviride* combined. The expression of 1993 genes was modulated by the presence of *A. mellea* and *T. atroviride* alone and combined (cluster 7), while that of 478 genes was affected by the presence of *A. mellea* or *T. atroviride* alone (cluster 8). In addition, six minor clusters comprised DEGs affected by 24 h incubation in the soil matrix, with specific profiles in the presence of *A. mellea* and/or *T. atroviride* (clusters from 9 to 14).

**Fig. 3:**
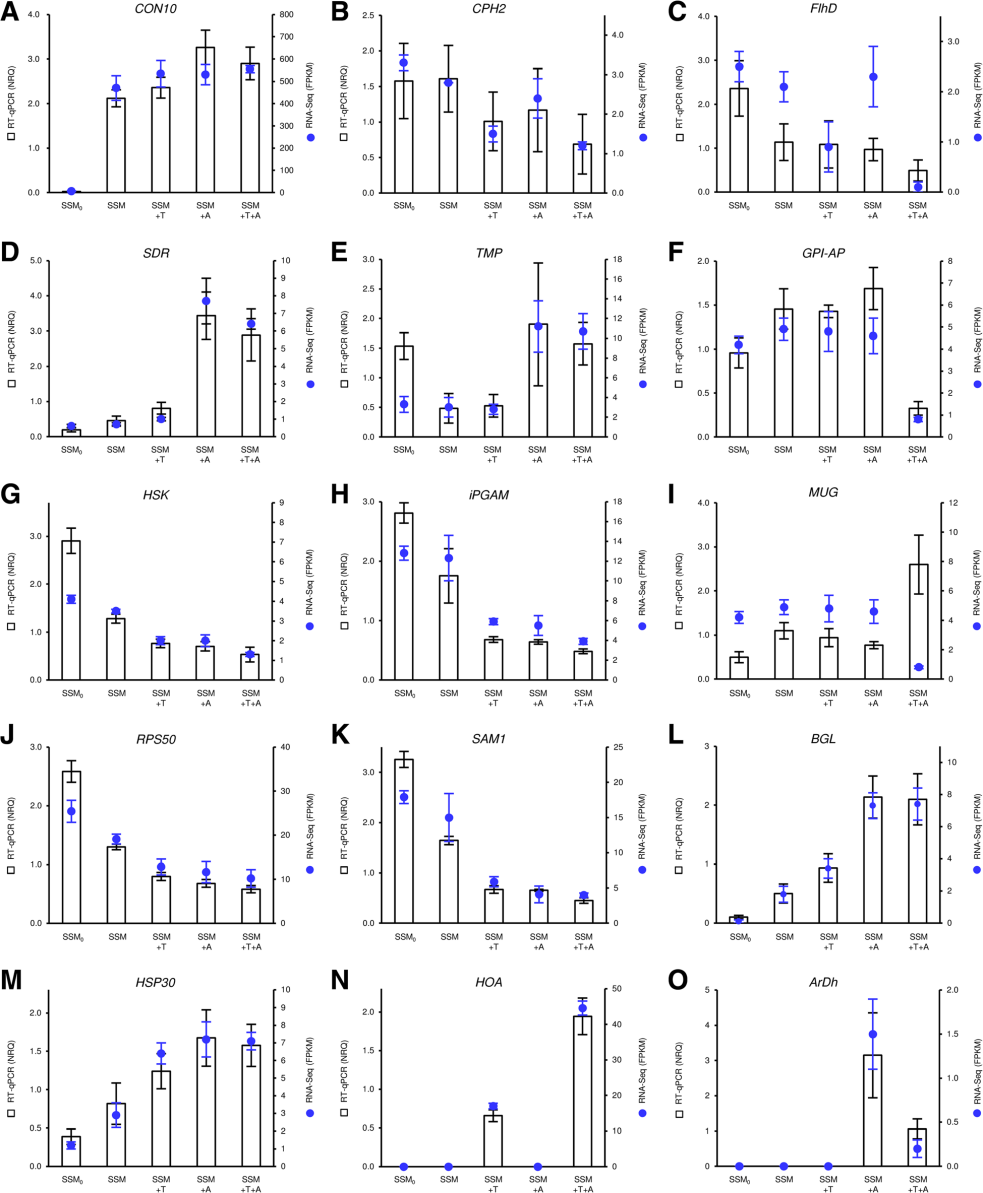
Comparison of RNA-Seq and real-time RT-PCR results. Expression profiles of **a** conidiation-specific protein 10 of *Aspergillus niger* (*CON10*), **b** transcription factor CPH2 of *Debaryomyces hansenii* (*CPH2*), **c** flagellar transcriptional regulator of *Cupriavidus metallidurans* (*FlhD*), **d** short-chain type dehydrogenase/reductase of *Pichia stipitis* (*SDR*), **e** transmembrane pair family protein of *Pseudomonas protegens* (*TMP*), **f** GPI-anchored protein 45 of *P. stipitis* (*GPI-AP*), **g** probable homoserine kinase of *Schizosaccharomyces pombe* (*HSK*), **h** 2,3-bisphosphoglycerate-independent phosphoglycerate mutase of *A. niger* (*iPGAM*), **i** meiosis upregulated gene UPF0590 *Penicillium chrysogenum* (*MUG*), **j** 50S ribosomal protein L14 of *Azorhizobium caulinodans* (*RPS50*), **k** S-adenosylmethionine synthase 1 of *Saccharomyces cerevisiae* (*SAM1*), **l** ß-glucosidase 4 of *Fusarium oxysporum* (*BGL*), **m** 30 kDa heat shock protein of *D. hansenii* (*HSP30*), **n** 4-hydroxy-2-oxovalerate aldolase of *Trichoderma atroviride* (*HOA*), **o** D-arabinitol dehydrogenase of *Armillaria mellea* (ArDH). *Blue dotted lines* represent expression levels (FPKM) calculated by RNA-Seq analysis and reported as means and standard errors of three replicates for each condition: the simplified soil microcosm collected at the beginning of the experiment (SSM_0_) and 24 h after incubation either without exogenous fungi (SSM), with the biocontrol agent *T. atroviride* (SSM+T), with the plant pathogen *A. mellea* (SSM+A) or with both (SSM+T+A). *White histograms* represent the relative expression levels calculated by quantitative real-time RT-PCR analysis as normalised relative quantities (NRQ;[52]) as compared to the mean expression level in all conditions, using *Actin 1* of *Schizosaccharomyces pombe* and *Elongation factor 1α* of *Fusarium oxysporum* as reference genes

To validate the RNA-Seq results, the expression levels of 15 DEGs were analysed by real-time RT-PCR (Fig. 3a–q). Genes with different expression profiles belonging to different soil microorganisms were analysed, including genes associated with different functional categories (Additional file 2). Although the extent of modulation revealed by real-time RT-PCR and RNA-Seq may differ [67, 68], the expression profiles obtained using the two methods agreed totally for ten genes. The expression profiles generated by real-time RT-PCR and RNA-Seq differed in one or all of the conditions for three (Fig. 3e, g and i) and two (Fig. 3d and c) genes, respectively. These differences could be due to differences in sensitivity, particularly in distinguishing members of multigene families and orthologous genes.

### Metabolic pathways differentially affected by *Armillaria mellea* and *Trichoderma atroviride*

The metabolic pathways of the simplified soil microcosm were affected overall by 24 h incubation in the soil matrix, with similar expression profiles in the presence or absence of the plant pathogen and biocontrol agent (clusters 1 and 15; Additional file 10A and B). Complex reprogramming of primary metabolic processes (organic acid, carbohydrate and lipid metabolism) indicated adaptation of the microbial communities, from the nutrient conditions of the rich microbiological media to the harsh habitat of the soil matrix (Additional file 11). For example, genes involved in the metabolism of aromatic compounds (quinate, vanillate, ferulate, and benzene metabolism) and in energy production under anaerobic conditions (formate and acetate metabolism) were upregulated by incubation in the soil matrix as possible adaptation of the simplified soil microcosm to humic acid metabolism under semi-anaerobic conditions. Likewise, growth regulators and conidiation-related genes were modulated by incubation of the simplified soil microcosm as a consequence of adaptation processes from in vitro to soil conditions (Additional file 7).

**Fig. 4:**
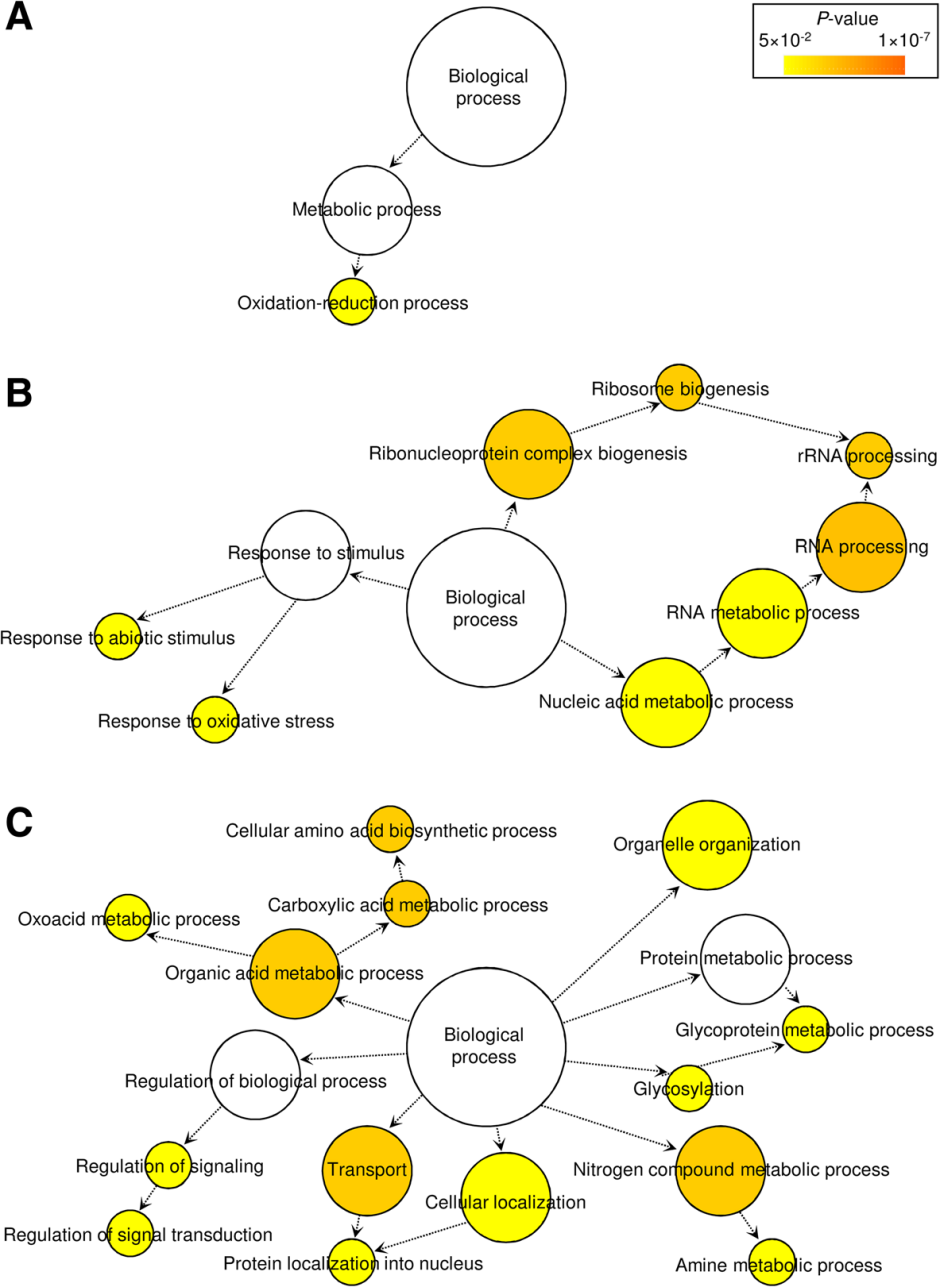
Biological networks of significantly enriched Gene Ontology (GO) terms. GO biological process terms of the simplified soil microcosm, upregulated by the presence of **a** *T. atroviride* (cluster 3), **b** *A. mellea* (cluster 5) or **c** both *A. mellea* and *T. atroviride* (cluster 7). Significantly enriched GO terms (*P* < 0.05) were identified using the BiNGO tool [42] and visualised with Cytoscape software [43]. The colour scale legend indicates the level of significance for enriched GO terms. White nodes indicate not significantly overrepresented categories. Dotted lines indicate connection between biological process categories in the GO chart, where ancestor and child are omitted for simplicity

The introduction of *T. atroviride* caused modulation of genes related to secondary metabolic pathways (metabolism of folate and toxic compounds) and regulators of cell growth and differentiation (controllers of meiotic processes and regulators of hyphal development) in the simplified soil microcosm (cluster 3; Additional file 10C). Oxidation-reduction processes (Fig. 4a), genes involved in signal transduction (kinases and phosphatases) and defence response (multidrug- and heavy metal- efflux proteins) were activated by the simplified soil microcosm as a reaction to *T. atroviride* (Additional file 7). Conversely, nucleic acid and RNA metabolic processes were mainly activated by the simplified soil microcosm during incubation with *A. mellea* (cluster 5), as well as processes related to response to stimuli (receptors and protein kinases) and to oxidative stresses (catalases and oxidoreductases; Fig. 4b, Additional files 7 and 10D).

The presence of both *A. mellea* and *T. atroviride* (cluster 7) upregulated expression regulators (transcriptional regulators NRG1 and NahR, a transcriptional activator of hydrogenase-4), genes implicated in defence processes (drug resistance proteins, protein KRE1, and ABC transporters for drug resistance) and secondary metabolism (Additional file 10E). Metabolisms of amino acids and amines were upregulated, as well as the regulation processes of signal transduction and protein localization (Fig. 4c). Conversely, genes related to regulation of cell division (spindle pole body-associated proteins, telomere length regulators, actin and myosin genes), cell cycle (mitotic cyclins, meiotic activators and cell division control proteins) and microbial growth (negative regulator of sporulation PMD1, morphogenesis-related gene MSB1, genes WHI4 and DSE2) were downregulated in the simplified soil microcosm (Additional file 7), as a possible consequence of competition with *A. mellea* and *T. atroviride*.

## Discussion

**Fig. 5:**
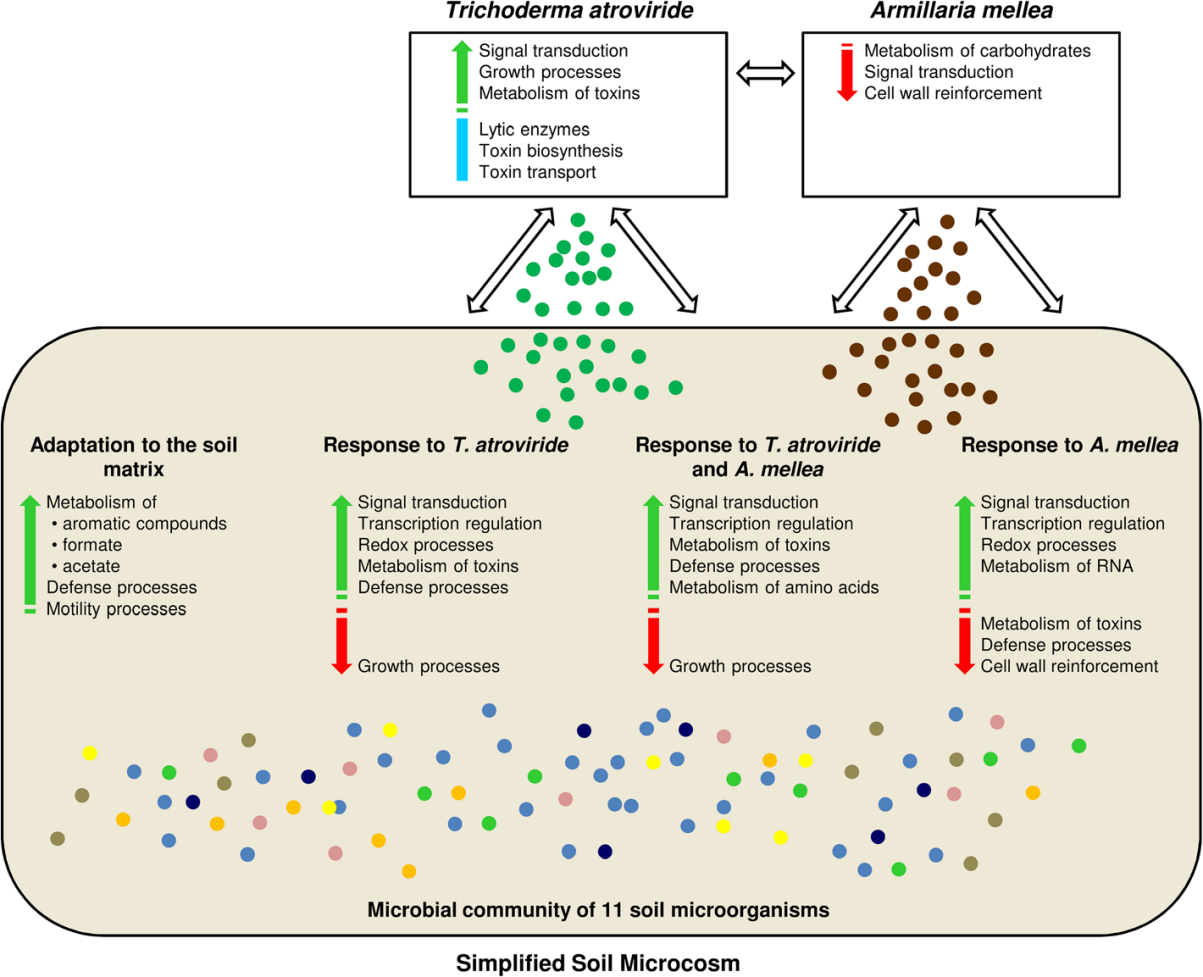
Overview of main processes modulated by the simplified soil microcosm, *Armillaria mellea* and *Trichoderma atroviride*. Main upregulated (*green arrow*) and downregulated (*red arrow*) processes are distinguished according to expression profiles: genes with similar expression in the presence or absence of *A. mellea* and *T. atroviride* (adaptation to the soil matrix, cluster 1); genes modulated by the presence of *T. atroviride* (response to *T. atroviride*, cluster 3), *A. mellea* (response to *A. mellea*, cluster 5) or both *A. mellea* and *T. atroviride* (response to *T. atroviride* and *A. mellea*, cluster 7). The *blue line* indicate overrepresented processes already expressed by *T. atroviride* in the simplified soil microcosm and not modulated by *A. mellea* introduction

Molecular interactions between biocontrol agents and target pathogens have mainly been studied in dual culture assays [2, 7, 8, 10, 18] and little is known about the more intricate molecular interplay of a complex microbial community. Microorganisms can sense and respond to the presence of neighbouring strains in the environment and alter their behaviour and performance accordingly [69]. In this study, we took advantage of RNA-Seq technology to study the interaction of a biocontrol agent (*T. atroviride*) with a ubiquitous plant pathogen (*A. mellea*) in a simplified soil microcosm containing 11 microorganisms. Complex microbial responses were found in the simplified soil microcosm. In particular, major transcriptional regulation occurred when single colonies grown on rich media in Petri dishes were transferred to the soil matrix, reflecting microbial acclimatisation to a different environment and the presence of other members of the microbial community. However, specific molecular responses were activated by the simplified soil microcosm in response to the introduction of the plant pathogen or the biocontrol agent, upregulating neutral adaptation processes and active defence mechanisms, respectively (the overview of main processes is reported in Fig. 5 and key DEGs are summarised in Additional file 12).

The results confirmed that RNA-Seq was an appropriate method for specifically estimating gene expression levels within a complex microbial consortia [33, 34]. However, the use of unambiguously mapped read pairs for gene expression quantification maximises specificity in the sequence count assignment, but could underestimate the expression levels of homologous genes within the microbial community. Moreover, gene expression levels in metatranscriptomic analysis are determined not only by transcriptional regulation, but also by the biomass of each microorganism, compared with other members of the community [36]. For example, high and low proportions of *P. protegens* and *B. subtilis* reads were obtained, respectively, in agreement with their respective biomass in the soil matrix. *S. pombe* had the same concentration as the mean of other soil microorganisms and accounted for the greatest proportion of sequenced read pairs, resulting the most transcriptionally active microorganisms of the simplified soil microcosm.

### Metabolic adaptation of the simplified soil microcosm in the soil matrix was not modified by the introduction of *Armillaria mellea* and *Trichoderma atroviride*

Major transcriptional changes of genes related to complex metabolic pathways occurred through incubation in the soil matrix and showed similar expression profiles in the presence or absence of *A. mellea* and *T. atroviride* (cluster 1; Additional file 12), suggesting more significant adaptation to the soil environment than to the microbial intruders. Metabolic shifts from the nutrient conditions of the rich microbiological media to the soil matrix were highlighted by the transcriptional activation of genes implicated in the metabolism of aromatic compounds, formate and acetate (Fig. 5) possibly involved in the catabolism of humic acid [70] and energy production under anaerobic conditions [71].

The expression of defence genes was activated by the soil microorganisms to compete with each other, such as six hydrolases of *A. niger*, *C. metallidurans* and *F. oxysporum,* responsible for the detoxification of epoxides [2], and three polyketide synthases (PKS) of *A. niger*, *P. stipitis* and *D. hansenii*, responsible for the biosynthesis of toxic molecules [5, 7]. The upregulation of genes encoding 23 flagellar proteins, two flagellar regulators, three chemotaxis proteins and two pilus assembly proteins suggested the activation of bacterial cell motility, which could contribute to the initial phases of colonisation in the soil [72], as well as for exploreing soil niches and getting away from competitors [73]. Based on these results, metabolic adaptation and niche colonisation processes seem to be based on the metabolic plasticity and competition/defence capacity of each microorganism against neighbouring competitors in the soil matrix and these transcriptional changes dramatically increase the complexity of microbial community reprogramming.

### The simplified soil microcosm responded to the introduction of *Trichoderma atroviride* by activating defence-related processes

At transcriptional level, the overall response of the simplified soil microcosm to the introduction of *T. atroviride* (328 DEGs of cluster 3) revealed a reaction to an antagonistic intruder, such as the activation of signal transduction processes, metabolism of toxic compounds, oxidation-reduction and defence processes, as well as shifts in cell growth and differentiation (Fig. 5, Additional file 12). Specifically, the activation of genes implicated in signal transduction processes (five protein kinases, a protein phosphatase and a calcium-binding protein) and transcriptional regulation (the transcriptional regulatory proteins HoxA and PHO23, a RNA polymerase and a zinc finger domain protein) indicated recognition and reaction of the simplified soil microcosm to the biocontrol agent. The microbial response included the upregulation of genes encoding enzymes implicated in the metabolism of toxic compounds, such as a nonribosomal peptide synthetase (NRPS) of *A. niger*, a methenyltetrahydrofolate cyclohydrolase and a riboflavin biosynthesis protein of *A. caulinodans*, a formylbenzenesulfonate dehydrogenase and a phytoene synthase of *C. metallidurans*, and an isovaleryl dehydrogenase of *F. oxysporum*. Furthermore, other defence-related genes were upregulated, possibly to protect the soil microbial community against the biocontrol agent, such as three resistance proteins and a gallate transporter of *C. metallidurans*, a peptidase of *P. protegens* and a protease of *P. stipitis*. Proteases and enzymes responsible for the detoxification of toxic molecules have already been found to be transcribed in the plant pathogenic fungi *Rhizoctonia solani* and *A. niger* in response to antagonistic strains belonging to *Bacillus* and *Serratia* genera [2, 5], suggesting that biocontrol agents activate typical defence processes in neighbour microorganisms. Oxidoreductases of *A. niger* and *F. oxysporum* were upregulated in the simplified soil microcosm by *T. atroviride* to remove active oxidants, as already observed for *R. solani* in response to *Serratia* spp. [2]. Conversely, stress-related genes were downregulated in *A. niger* in response to *B. subtilis* attachment [5], suggesting that microbial responses could differ in mutualistic and antagonistic interactions.

Biocontrol agents actively compete with indigenous microorganisms through the production of antimicrobial compounds [74] and *T. atroviride* can antagonize and/or parasitize target fungi [16], suggesting that this fungal strain may have inhibited the growth of the soil microbial community. This hypothesis is in line with the inhibition of growth and differentiation processes of the simplified soil microcosm by *T. atroviride* introduction that was suggested by the downregulation of six regulators of cell division and four genes implicated in hyphal growth of *A. niger*, *D. hansenii*, *F. oxysporum*, *S. cerevisiae* and *S. pombe*. Likewise, a regulator of sporulation and two genes related to cell wall biogenesis were downregulated in *D. hansenii* and *S. cerevisiae*. Transcriptional changes of the growth- and morphology-related genes of plant pathogens in response to antagonistic bacteria are known to be linked to stress responses [2], and they could be proposed as markers of competitive interactions among the microbiota. Two flagellar transcriptional regulators (Rmet_1690 and Rmet_1691) were downregulated in response to *T. atroviride* introduction, and chemotactic motility genes were modulated in *Collimonas fungivorans* and *Serratia plymuthica* incubated with *A. niger* [8] and *R. solani* [10], respectively. Taken together, these expression profiles reflected the reaction of the simplified soil microcosm to the competition displayed by the biocontrol agent *T. atroviride* through antagonism and production of toxic molecules.

### The simplified soil microcosm responded to the introduction of *Armillaria mellea* by activating neutral adaptation processes

The transcriptional response of the simplified soil microcosm to *A. mellea* introduction (1203 DEGs of cluster 5) activated more neutral adaptation processes as compared to *T. atroviride* introduction (Fig. 5, Additional file 12). Six protein kinases, two receptors and 21 transcription regulators were upregulated in the simplified soil microcosm and are candidate activators of the microcosm response to *A. mellea*. Similar expression changes were found for 16, 14 and 13 genes responsible for RNA metabolism, ribosome biogenesis and regulation of oxidative stress, respectively. Signal transduction [7] and oxidation-reduction processes [2] are commonly activated during microbe-microbe communications and expression profiles reflect the response of the simplified soil microcosm to *A. mellea*. However, genes implicated in defence-related processes were mainly down- and up-regulated in response to *A. mellea* and to *T. atroviride*, respectively*.* These opposite regulations indicate that the simplified soil microcosm may recognise the antagonistic properties of *T. atroviride* and react with early activation of defence processes, which are not required in the presence of the plant pathogen. Specifically, the *A. mellea* introduction downregulated the expression of genes responsible for cell wall reinforcement, stress-related processes and the metabolism of toxic and aromatic compounds in *A. niger*, *A. caulinodans*, *C. metallidurans*, *P. chrysogenum*, *S. cerevisiae* and *S. pombe*. In nature microbes have evolved mechanisms not only to fight, but also to adapt, and in some cases to support each other [5]. The microbial transcriptional response differs strongly during antagonistic, neutral and beneficial interactions [75] and defence-related processes are generally downregulated during mutualistic interactions [5]. Therefore, the simplified soil microcosm was probably able to recognise *A. mellea* as a non-competitive intruder and it did not stimulate early antagonistic reactions, but rather neutral adaptation processes to this exogenous fungus. However, microbial communities of suppressive soils could activate antagonistic traits against soil phytopathogens after long incubation periods [6].

### The response mechanisms to *Trichoderma atroviride* dominated over those to *Armillaria mellea* following simultaneous introduction to the simplified soil microcosm

When *A. mellea* and *T. atroviride* were simultaneously introduced to the simplified soil microcosm (cluster 7), the activation of defence-related genes indicated an attempted defence response by the microbial community, mediated by the upregulation of genes implicated in cell wall reinforcement, lytic processes (proteases and peptidases) and drug resistance (Fig. 5, Additional file 12). Secondary metabolic processes play a crucial role in the biocontrol activities of *T. atroviride* [76], and genes for the metabolism of aromatic compounds and biofilm formation were upregulated, possibly to defend the microbial community against this fungal intruder. Likewise, two ABC transporter genes were upregulated in *A. caulinodans* and *C. metallidurans*, and their activation has been associated with detoxification pathways during biocontrol processes [76]. Increased protein synthesis during biocontrol interactions was linked to the defence mechanisms of the prey microorganisms [77], and genes implicated in the metabolism of amino acids were upregulated by the simplified soil microcosm. However, microbial distress was indicated by the downregulation of growth-related genes, such as 32 regulators of division processes, 31 regulators of the cell cycle, ten genes implicated in DNA replication processes and nine in chromatin remodelling processes.

In addition to defence processes, signal transduction and transcription regulations were also activated. In particular, genes encoding protein kinase, protein phosphatases and signalling proteins were activated in *C. metallidurans*, *D. hansenii*, *F. oxysporum*, *P. chrysogenum*, *P. protegens* and *P. stipitis*. Likewise, the upregulation of 23 transcription factors and 22 genes implicated in the RNA metabolism reflected the response of the microbial community. Specifically, the upregulated transcription factors *prtT* (Ps_47947), *YjiE* (Rmet_0397), *NRG1* (Ps_32504) and *NahR* (Rmet_0894) are possibly implicated in the regulation of defence processes, such as protease biosynthesis, response to environmental stress [78], regulation of biofilm formation [79] and secondary metabolism [80], respectively. Although different genes were implicated, key functional processes modulated by the simplified soil microcosm in response to the simultaneous introduction of *A. mellea* and *T. atroviride* revealed expression profiles similar to those for the condition in which only *T. atroviride* was introduced. The response to the antagonistic fungus may therefore dominate neutral reactions to the less competitive plant pathogen *A. mellea*. However, 180 genes with an unknown function were upregulated, indicating that several yet-to-be identified proteins may have been involved in the response of the simplified soil microcosm.

### *Trichoderma atroviride* biocontrol processes were already activated by the simplified soil microcosm and *Armillaria mellea* did not expresses antagonistic traits

*T. atroviride* DEGs included 30 and 58 genes up and down-regulated during incubation in the presence of *A. mellea*, respectively (cluster 17). In particular, upregulated genes of calcium and cAMP-mediated signalling were possibly implicated in the response of *T. atroviride* to *A. mellea* (Fig. 5, Additional file 12). The activation of two and five genes related to growth regulation and metabolic processes, respectively suggest stimulation of *T. atroviride* growth in the simplified soil microcosm containing *A. mellea*, in agreement with the transcriptional response during mycoparasitic interaction with *R. solani* [81]. Genes implicated in the metabolism of aromatic compounds were upregulated (a 6-hydroxy-D-nicotine oxidase, a 3-hydroxybenzoate 6-hydroxylase and a 4-hydroxy-2-oxovalerate aldolase) and they are possibly involved in the catabolism of microbial compounds. A *T. atroviride* peptidase was upregulated and is potentially implicated in biocontrol activity against *A. mellea*. Key lytic enzymes in biocontrol activity against fungal plant pathogens [76, 82, 83] were already expressed by *T. atroviride* during incubation in the simplified soil microcosm (SSM+T sample), and they were not further modulated by *A. mellea* introduction (SSM+T+A sample), as in the case of 23 proteases, 13 ß-glucanases, ten chitinases and two glucosaminidases. Likewise, two PKSs, eight NRPSs, 17 enzymes of toxin metabolism and four of terpenoid metabolism (terpene, trichothecene and trichodiene), six siderophore transporters and 23 ABC transporters possibly implicated in the production of toxic biocontrol molecules and antifungal components [84, 85] were highly expressed by *T. atroviride* in the simplified soil microcosm. These expression profiles suggested that biocontrol processes were already activated by *T. atroviride* incubated in the soil matrix to compete with soil microorganisms and the simplified soil microcosm consequently reacted by activating defence processes and detoxification mechanisms. On the other hand, the expression of genes encoding lytic enzymes and toxic secondary metabolites was underrepresented in *A. mellea* as compared with *T. atroviride*. Specifically, only 12 proteases, seven ß-glucanases, eight chitinases were expressed by *A. mellea* during incubation in the simplified soil microcosm in the presence (SSM+T+A) or absence (SSM+A sample) of *T. atroviride* and no PKSs, NRPSs and genes of siderophore transport and toxin efflux were expressed (Additional file 12), supporting that the activation of defence mechanisms by the simplified soil microcosm was not needed in response to this non-competitive intruder. Moreover, *A. mellea* genes responsible for sugar transport, energy and carbohydrate metabolism were downregulated in response to *T. atroviride* introduction, possibly reflecting the inhibition of *A. mellea* growth in the presence of the biocontrol agent. Likewise, genes implicated in signal transduction processes (a calmodulin and a Ras-related protein) and cell wall reinforcement were downregulated, suggesting that no appropriate cellular reactions were activated by *A. mellea* in response to *T. atroviride* in the simplified soil microcosm.

## Conclusions

This transcriptome analysis of a simplified soil microcosm is a useful approach to model the complex microbial responses of a microbial community. The change in the growing environment, including both the nutrient-limiting conditions of the soil matrix and the presence of individuals of other species, profoundly impact the transcriptome and the metabolic plasticity of the microbial community. The transcriptional responses of the microbial community differed according to the habitus of microbial intruders (plant pathogen or biocontrol agent). The two intruders were specifically recognised by the other members of the simplified soil microcosm: the biocontrol agent stimulated early defence reactions in the soil microbiota, while the plant pathogen seemed to be perceived as a non-competitive intruder, resulting in the downregulation of defence mechanisms. This opposite response of the simplified soil microcosm agreed with the stronger expression of lytic enzymes and antimicrobial pathways by the biocontrol agent as compared with the plant pathogen. Key biocontrol processes of *T. atroviride* were already activated, most probably by the other microorganisms of the simplified soil microcosm, and they were not further enhanced by *A. mellea* introduction, in agreement with the generic antagonistic properties of *T. atroviride* rather than specific activity against *A. mellea*. These results represent a step towards understanding the molecular interplay underlying interactions among soil microorganisms and the early transcriptional impact of biocontrol agents on non-target microorganisms. However, further functional and metabolomic studies with a broad range of different soil microorganisms and sampling time points are required in order to deeply characterize the recognition mechanisms of competitive and non-competitive intruders.

## Additional files

Additional file 1: Concentration of the soil microorganism in the simplified soil microcosm. (DOCX 18 kb)
Additional file 2: Primer sequences of the microcosm genes analysed by real-time RT-PCR. (XLSX 18 kb)
Additional file 3: RNA-Seq sequencing and mapping results for each replicate. (PDF 14 kb)
Additional file 4: Distribution of read pair alignments to the genomes of the soil microorganisms. (**A, B**) Distribution of read pair alignments to the 13 soil microorganisms calculated with the Samtools software [30] and expressed as a percentage (%) of total alignments to the microcosm genome. (**C, D**) Distribution of unique read pairs mapping to genes of the 13 soil microorganisms, counted using HTSeq [32] and expressed as a percentage (%) of total unique read pairs mapping to genes in the microcosm genome. (**E, F**) Percentage (%) of expressed genes (more than one read pair) calculated as compared to the total predicted genes for each soil microorganism. Mean and standard error values of three replicates are reported for each condition: the simplified soil microcosm collected at the beginning of the experiment (SSM_0_) and 24 h after incubation either without exogenous fungi (SSM), with the biocontrol agent *Trichoderma atroviride* (SSM+T), with the plant pathogen *Armillaria mellea* (SSM+A) or with both (SSM+T+A). (PDF 23 kb)
Additional file 5: Expression levels of genes of the simplified soil microcosm. (XLSX 39274 kb)
Additional file 6: Pearson’s correlation coefficients among replicates and conditions for RNA-Seq analysis. (PDF 6 kb)
Additional file 7: Clustering and functional annotation results of differentially expressed genes. (XLSX 15048 kb)
Additional file 8: Proportion of expressed and differentially expressed genes for each soil microorganism. (PDF 8 kb)
Additional file 9: Distribution of differentially expressed genes of each soil microorganism in 18 clusters, based on the expression profiles. (XLSX 12 kb)
Additional file 10: Metabolic pathways of the simplified soil microcosm, modulated by incubation in the soil matrix. Metabolic pathways deactivated (left panels) and activated (right panels) by 24 h incubation in the soil matrix (**A**) without reinforced modulation (cluster 1) and (**B**) with reinforced modulation (cluster 15) in the presence of *Armillaria mellea* and *Trichoderma atroviride* combined. Metabolic pathways modulated by the introduction of (**C**) *T. atroviride* (cluster 3), (**D**) *A. mellea* (cluster 5), or (**E**) both (cluster 7). KEGG pathways were visualised using the iPath2 tool [48], the pathways of upregulated (green) and downregulated (red) genes were highlighted, and a section of the most relevant pathways is reported for each panel. (PDF 2815 kb)
Additional file 11: Biological networks of Gene Ontology (GO) terms. GO biological process terms of the simplified soil microcosm, upregulated by incubation in the soil matrix with similar expression profiles in the presence or absence of *Armillaria mellea* and *Trichoderma atroviride* (cluster 1). Significantly enriched GO terms (*P* < 0.001) were identified using the BiNGO tool [42] and visualised with Cytoscape software [43]. The colour scale legend indicates the level of significance for enriched GO terms. White nodes indicate not significantly overrepresented categories. (PDF 111 kb)
Additional file 12: Key differentially expressed genes discussed in the manuscript, based on their functional categories and expression profiles. Each sheet contains the genes in each cluster discussed. (XLSX 23094 kb)

## Abbreviations

CFUs: Colony forming units
Ct: Threshold cycle
DEGs: Differentially expressed genes
Eff: Efficiency of the quantitative real-time PCR
FDR: False discovery rate
FPKM: Fragments per kilobase per million of unambiguously mapped read pairs
GO: Gene Ontology
KAAS: KEGG pathways using the KEGG Automatic Annotation Server
LB: Luria-Bertani broth
MEA: Malt extract agar
NRPS: Nonribosomal peptide synthetase
OD: Optical density
PDA: Potato dextrose agar
PKS: Polyketide synthases
RQ: Relative quantities
RT-qPCR: Quantitative real-time PCR
SSM: Simplified soil microcosm incubated for 24 h either without exogenous fungi
SSM+A: Simplified soil microcosm incubated for 24 h with the plant pathogen *Armillaria mellea*
SSM+T: Simplified soil microcosm incubated for 24 h with the biocontrol agent *Trichoderma atroviride*
SSM+T+A: Simplified soil microcosm incubated for 24 h with the biocontrol agent *T. atroviride* and the plant pathogen *A. mellea*
SSM0: Simplified soil microcosm incubated collected at the beginning of the experiment just after inoculation of the 11 soil microorganisms
