# Supplementary material for: Transcriptomic responses of a simplified soil microcosm to a plant pathogen and its biocontrol agent reveal a complex reaction to harsh habitat
Source: BMC Genomics. 2016 Oct 27;17:838. doi: 10.1186/s12864-016-3174-4 (PMC5081961; doi:10.1186/s12864-016-3174-4)
Supplement: Additional file 1: — Concentration of the soil microorganism in the simplified soil microcosm. (DOCX 18 kb) [file 12864_2016_3174_MOESM1_ESM.docx]

**Additional file 1.** Concentration of the soil microorganism in the simplified soil microcosm.

| **Microbial strain** | **Type^a^** | **Medium^b^** | **Concentration in the simplified soil microcosm (CFUs g^-1^)^c^** | |
| --- | --- | --- | --- | --- |
| *Azorhizobium caulinodans* ORS 571 | B | LB | 1.31 ± | 0.04 × 10^7^ |
| *Bacillus subtilis* subsp. *subtilis* 168 | B | LB | 2.07 ± | 0.47 × 10^5^ |
| *Cupriavidus metallidurans* CH34 | B | LB | 1.98 ± | 0.02 × 10^7^ |
| *Pseudomonas protegens* Pf-5 | B | LB | 2.27 ± | 0.33 × 10^7^ |
| *Debaryomyces hansenii* CBS767 | Y | MEA | 6.00 ± | 0.10 × 10^5^ |
| *Pichia stipitis* CBS 6054 | Y | MEA | 2.47 ± | 0.55 × 10^6^ |
| *Saccharomyces cerevisiae* S288c | Y | MEA | 6.27 ± | 0.57 × 10^6^ |
| *Schizosaccharomyces pombe* 972h | Y | MEA | 3.07 ± | 0.58 × 10^6^ |
| *Aspergillus niger* CBS 513.88 | FF | PDA | 2.07 ± | 0.18 × 10^6^ |
| *Fusarium oxysporum* f. sp. *lycopersici* 4287 | FF | PDA | 5.80 ± | 0.50 × 10^6^ |
| *Penicillium chrysogenum* Wisconsin54-1255 | FF | PDA | 2.40 ± | 0.31 × 10^6^ |
| *Trichoderma atroviride* SC1 | BA | MEA | 8.80 ± | 0.42 × 10^5^ |
| *Armillaria mellea* M6132 | PP | MEA | 1.67 ± | 0.01 × 10^3^ |
|  |  |  |  |  |

^a^ Soil microorganisms are classified as: bacterium (B), yeast (Y), filamentous fungus (FF); biocontrol agent (BA), and plant pathogen (PP).

^b^ Culture media used to assess the concentration of the each microbial strain in the Luria-Bertani broth (LB), malt extract agar (MEA) and potato dextrose agar (PDA).

^c^ The number of colony forming units (CFUs) in the simplified soil microcosm (CFUs g^-1^) was determined with dilution plating method. Mean values and standard errors from three replicates are presented for each strain. CFUs of *Armillaria mellea* refer to the addition of 0.5 g of ground mycelium of the fungus.

No significant changes (t-test, *P* > 0.05) on microbial CFUs were found for each soil microorganism after single incubation in the soil matrix for 24 h at 25°C.
