# Supplementary material for: Transcriptomic responses of a simplified soil microcosm to a plant pathogen and its biocontrol agent reveal a complex reaction to harsh habitat
Source: BMC Genomics. 2016 Oct 27;17:838. doi: 10.1186/s12864-016-3174-4 (PMC5081961; doi:10.1186/s12864-016-3174-4)
Supplement: Additional file 3: — RNA-Seq sequencing and mapping results for each replicate. (PDF 14 kb) [file 12864_2016_3174_MOESM3_ESM.pdf]

Additional file 3. RNA-Seq sequencing and mapping results for each replicate.

Condition[a]	Replicate	Sequenced read pairs[b]	Filtered read pairs (%)[c]		Mapped reads (%)[d]		Unique read pairs (%)[e]		Multi read pairs (%)[f]		Unique read pairs mapping to genes (%)[g]	
SSM_0	1	54,867,635	46,512,002	(85)	45,466,335	(98)	10,442,923	(23)	3,5023,412	(77)	8,981,515	(86)
	2	53,137,108	44,843,649	(84)	43,827,359	(98)	11,547,703	(26)	3,2279,656	(74)	10,025,907	(87)
	3	47,288,179	41,240,720	(87)	40,375,071	(98)	10,878,932	(27)	2,9496,139	(73)	9,482,541	(87)
SSM_0	1	53,923,704	45,334,672	(84)	44,120,949	(97)	8,078,247	(18)	3,6042,702	(82)	6,228,246	(77)
	2	57,625,098	48,992,138	(85)	47,848,571	(98)	12,114,995	(25)	3,5733,576	(75)	9,893,233	(82)
	3	47,957,873	42,184,246	(88)	41,009,367	(97)	10,371,301	(25)	3,0638,066	(75)	8,358,839	(81)
SSM+T	1	53,770,221	47,001,494	(87)	45,259,937	(96)	10,679,232	(24)	3,4580,705	(76)	8,739,437	(82)
	2	74,788,948	66,797,521	(89)	64,626,032	(97)	14,367,754	(22)	5,0258,278	(78)	11,619,984	(81)
	3	60,209,616	50,657,014	(84)	48,771,238	(96)	11,850,153	(24)	3,6921,085	(76)	9,648,304	(81)
SSM+A	1	45,543,588	40,539,838	(89)	39,071,436	(96)	7,318,891	(19)	3,1752,545	(81)	5,676,838	(78)
	2	47,244,943	39,689,851	(84)	38,478,021	(97)	8,622,139	(22)	2,9855,882	(78)	6,919,365	(80)
	3	54,715,166	46,315,044	(85)	44,902,907	(97)	8,531,929	(19)	3,6370,978	(81)	6,710,241	(79)
SSM+T+A	1	51,885,907	43,582,841	(84)	41,864,067	(96)	8,676,131	(21)	3,3187,936	(79)	6,847,827	(79)
	2	80,245,613	71,084,866	(89)	68,440,718	(96)	14,731,717	(22)	5,3709,001	(78)	11,857,496	(81)
	3	47,733,908	41,138,248	(86)	39,542,785	(96)	8,746,714	(22)	3,0796,071	(78)	7,070,119	(81)

[a] Five different conditions were analysed in triplicate (named from 1 to 3) by RNA-Seq: the simplified soil microcosm collected at the beginning of the experiment (SSM_0) and 24 h after incubation either without exogenous fungi (SSM), with the biocontrol agent *Trichoderma atroviride* (SSM+T), with the plant pathogen *Armillaria mellea* (SSM +A) or with both (SSM+T+A).

[b] Read pairs of 100 nucleotides were obtained by the RNA-seq protocol followed by Illumina sequencing.

[c] Read pairs passing the quality check and the corresponding percentage (%) of the sequenced read pairs.

[d] Read pairs mapped to the microcosm genome and the corresponding percentage (%) of filtered read pairs.

[e] Read pairs unambiguously mapped to unique locations (unique read pairs) to the microcosm genome and the corresponding percentage (%) of total read pairs mapped to the microcosm genome.

[f] Read pairs mapped to more than one location (multi read pairs) to the microcosm genome and the corresponding percentage (%) of total read pairs mapped to the microcosm genome.
