# Supplementary material for: Transcriptomic responses of a simplified soil microcosm to a plant pathogen and its biocontrol agent reveal a complex reaction to harsh habitat
Source: BMC Genomics. 2016 Oct 27;17:838. doi: 10.1186/s12864-016-3174-4 (PMC5081961; doi:10.1186/s12864-016-3174-4)
Supplement: Additional file 4: — Distribution of read pair alignments to the genomes of the soil microorganisms. (A, B) Distribution of read pair alignments to the 13 soil microorganisms calculated with the Samtools software [30] and expressed as a percentage (%) of total alignments to the microcosm genome. (C, D) Distribution of unique read pairs mapping to genes of the 13 soil microorganisms, counted using HTSeq [32] and expressed as a percentage (%) of total unique read pairs mapping to genes in the microcosm genome. (E, F) Percentage (%) of expressed genes (more than one read pair) calculated as compared to the total predicted genes for each soil microorganism. Mean and standard error values of three replicates are reported for each condition: the simplified soil microcosm collected at the beginning of the experiment (SSM0) and 24 h after incubation either without exogenous fungi (SSM), with the biocontrol agent Trichoderma atroviride (SSM+T), with the plant pathogen Armillaria mellea (SSM+A) or with both (SSM+T+A). (PDF 23 kb) [file 12864_2016_3174_MOESM4_ESM.pdf]

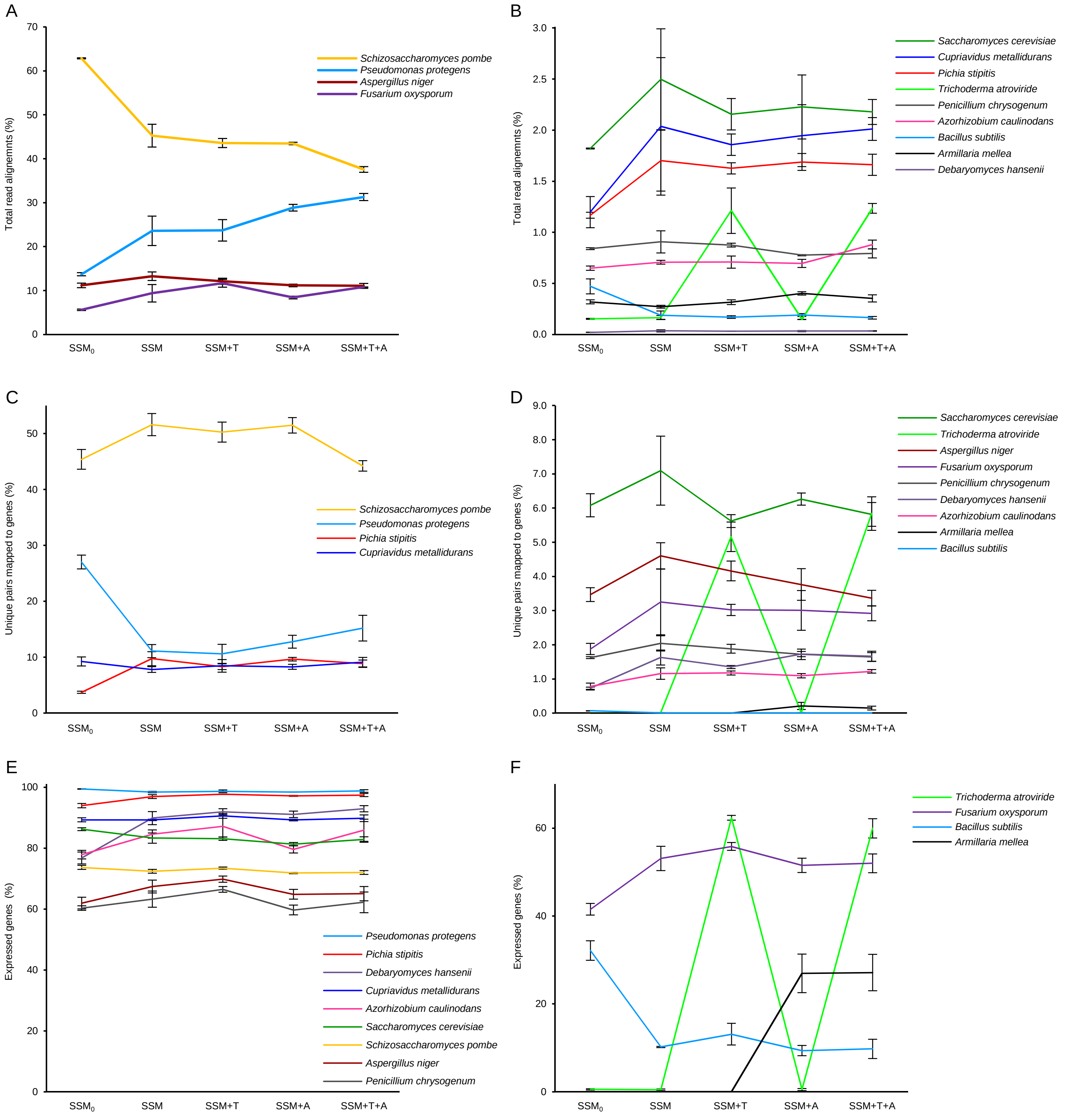


**Additional file 4** Distribution of read pair alignments to the genomes of the soil microorganisms. (**A, B**) Distribution of read pair alignments to the 13 soil microorganisms calculated with the Samtools software [27] and expressed as a percentage (%) of total alignments to the microcosm genome. (**C, D**) Distribution of unique read pairs mapping to genes of the 13 soil microorganisms, counted using HTSeq [29] and expressed as a percentage (%) of total unique read pairs mapping to genes in the microcosm genome. (**E, F**) Percentage (%) of expressed genes (more than one read pair) calculated as compared to the total predicted genes for each soil microorganism. Mean and standard error values of three replicates are reported for each condition: the simplified soil microcosm collected at the beginning of the experiment ($SSM_0$) and 24 h after incubation either without exogenous fungi (SSM), with the biocontrol agent
