# Supplementary material for: Transcriptomic responses of a simplified soil microcosm to a plant pathogen and its biocontrol agent reveal a complex reaction to harsh habitat
Source: BMC Genomics. 2016 Oct 27;17:838. doi: 10.1186/s12864-016-3174-4 (PMC5081961; doi:10.1186/s12864-016-3174-4)
Supplement: Additional file 6: — Pearson’s correlation coefficients among replicates and conditions for RNA-Seq analysis. (PDF 6 kb) [file 12864_2016_3174_MOESM6_ESM.pdf]

Additional file 6. Pearson's correlation coefficients among replicates and conditions for RNA-Seq analysis.

**Condition**[a]	**Correlations among replicates**[b]		**Correlations among conditions**[b]				
	**2nd replicate**	**3rd replicate**	**SSM_0**	**SSM**	**SSM+T**	**SSM+A**	**SSM+T+A**
SSM_0	0.968	0.989	1				
SSM	0.996	0.963	0.854	1			
SSM+T	0.997	0.995	0.855	0.982	1		
SSM+A	0.989	0.994	0.875	0.992	0.983	1	
SSM+T+A	0.990	0.986	0.877	0.975	0.969	0.987	1

[a] Five different conditions were analyzed in triplicate (named from 1 to 3) by RNA-Seq: the simplified soil microcosm collected at the beginning of the experiment (SSM_0) and 24 h after incubation either without exogenous fungi (SSM), with the biocontrol agent *Trichoderma atroviride* (SSM+T), with the plant pathogen *Armillaria mellea* (SSM +A) or with both (SSM+T+A).

[b] Pearson's correlations between the first replicate and the second or the third replicates were calculated for each condition based on the expression levels of all genes of the simplified soil microcosm (P = 0.00001).

[b] Pairwise Pearson's correlations among the five different conditions of the simplified soil microcosm were calculated based on the mean expression levels of all genes (P = 0.00001).
