# Supplementary material for: Transcriptomic responses of a simplified soil microcosm to a plant pathogen and its biocontrol agent reveal a complex reaction to harsh habitat
Source: BMC Genomics. 2016 Oct 27;17:838. doi: 10.1186/s12864-016-3174-4 (PMC5081961; doi:10.1186/s12864-016-3174-4)
Supplement: Additional file 8: — Proportion of expressed and differentially expressed genes for each soil microorganism. (PDF 8 kb) [file 12864_2016_3174_MOESM8_ESM.pdf]

Additional file 8. Proportion of expressed and differentially expressed genes for each soil microorganism.

**Microbial strain**	**Genes**[a]	**Expressed genes (%)**[b]		**Differentially expressed genes (%)**[c]	
Azorhizobium caulinodans ORS 571	4,781	4,166	(87)	993	(24)
Bacillus subtilis subsp. *subtilis* 168	4,371	1,406	(32)	175	(12)
Cupriavidus metallidurans CH34	6,829	6,187	(91)	2,333	(38)
Pseudomonas protegens Pf-5	6,212	6,177	(99)	5,093	(82)
Debaryomyces hansenii CBS767	6,272	5,830	(93)	2,776	(48)
Pichia stipitis CBS 6054	5,807	5,674	(98)	3,645	(64)
Saccharomyces cerevisiae S288c	7,126	6,145	(86)	2,640	(43)
Schizosaccharomyces pombe 972h	7,018	5,171	(74)	1,238	(24)
Aspergillus niger CBS 513.88	14,445	10,087	(70)	3,217	(32)
Fusarium oxysporum f. sp. *lycopersici* 4287	18,066	10,085	(56)	4,043	(40)
Penicillium chrysogenum Wisconsin54-1255	13,671	9,088	(66)	2,156	(24)
Trichoderma atroviride IMI 206040 [d]	11,863	7,398	(62)	88	(1)
Armillaria mellea DSM 3731 [e]	14,473	3,926	(27)	8	(0)

[a] Number of genes of the reference genome (genome databases are reported in Table 1).

[b] Mean number of expressed genes in at least one condition and the corresponding percentage (%) of total genes in the reference genome.

[c] Number of differentially expressed genes and the corresponding percentage (%) of expressed genes. The Pearson's correlation coefficient between numbers of expressed and differentially expressed genes was 0.53 (P = 0.06).

[d] The reference genome of *Trichoderma atroviride* IMI 206040 was used to align read pairs derived from *Trichoderma atroviride* SC1.

[e] The reference genome of *Armillaria mellea* DSM 3731 was used to align read pairs derived from *Armillaria mellea* M6132.
