# Supplementary material for: Transcriptomic responses of a simplified soil microcosm to a plant pathogen and its biocontrol agent reveal a complex reaction to harsh habitat
Source: BMC Genomics. 2016 Oct 27;17:838. doi: 10.1186/s12864-016-3174-4 (PMC5081961; doi:10.1186/s12864-016-3174-4)
Supplement: Additional file 10: — Metabolic pathways of the simplified soil microcosm, modulated by incubation in the soil matrix. Metabolic pathways deactivated (left panels) and activated (right panels) by 24 h incubation in the soil matrix (A) without reinforced modulation (cluster 1) and (B) with reinforced modulation (cluster 15) in the presence of Armillaria mellea and Trichoderma atroviride combined. Metabolic pathways modulated by the introduction of (C) T. atroviride (cluster 3), (D) A. mellea (cluster 5), or (E) both (cluster 7). KEGG pathways were visualised using the iPath2 tool [48], the pathways of upregulated (green) and downregulated (red) genes were highlighted, and a section of the most relevant pathways is reported for each panel. (PDF 2815 kb) [file 12864_2016_3174_MOESM10_ESM.pdf]

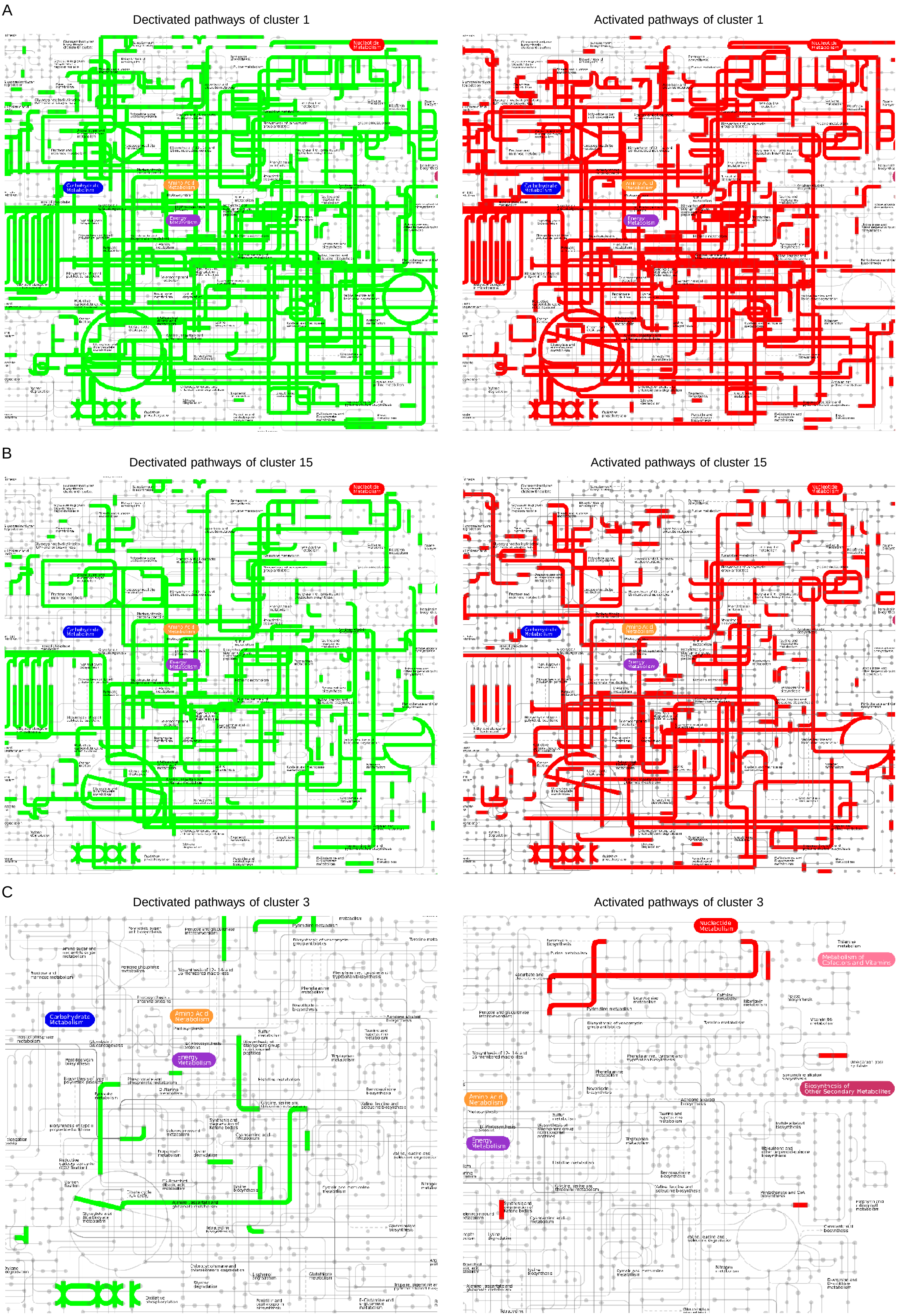
A
Dectivated pathways of cluster 1
Activated pathways of cluster 1
B
Dectivated pathways of cluster 15
Activated pathways of cluster 15
C
Dectivated pathways of cluster 3
Activated pathways of cluster 3

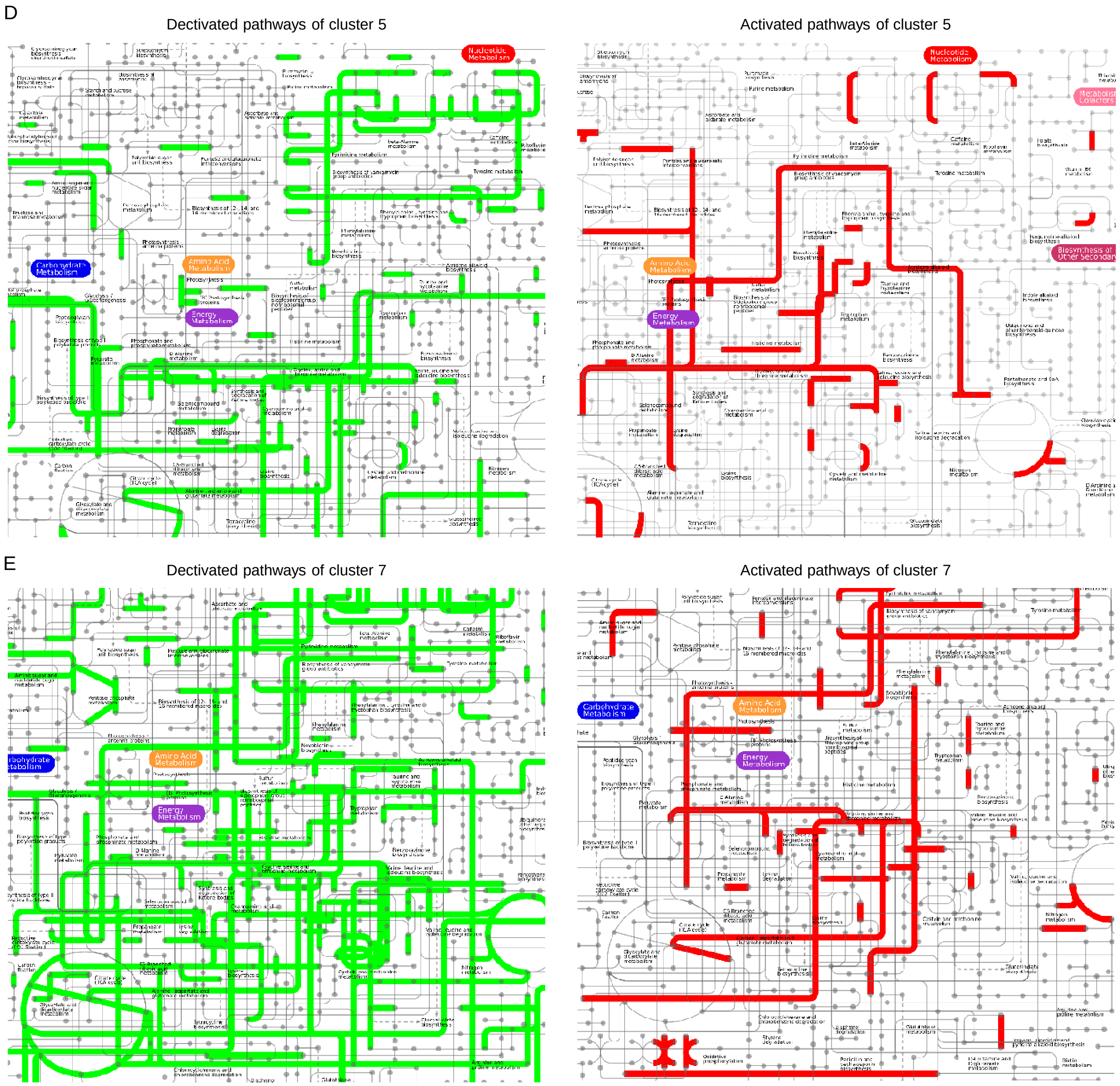

Additional file 10. Metabolic pathways of the simplified soil microcosm, modulated by incubation in the soil matrix. Metabolic pathways deactivated (left panels) and activated (right panels) by 24 h incubation in the soil matrix (**A**) without reinforced modulation (cluster 1) and (**B**) with reinforced modulation (cluster 15) in the presence of *Armillaria mellea* and *Trichoderma atroviride* combined. Metabolic pathways modulated by the introduction of (**C**) *T. atroviride* (cluster 3), (**D**) *A. mellea* (cluster 5), or (**E**) both (cluster 7). KEGG pathways were visualised using the iPath2 tool [45], the pathways of upregulated (green) and downregulated (red) genes were highlighted, and a section of the most relevant pathways is reported for each panel.
