# Supplementary material for: Transcriptomic responses of a simplified soil microcosm to a plant pathogen and its biocontrol agent reveal a complex reaction to harsh habitat
Source: BMC Genomics. 2016 Oct 27;17:838. doi: 10.1186/s12864-016-3174-4 (PMC5081961; doi:10.1186/s12864-016-3174-4)
Supplement: Additional file 11: — Biological networks of Gene Ontology (GO) terms. GO biological process terms of the simplified soil microcosm, upregulated by incubation in the soil matrix with similar expression profiles in the presence or absence of Armillaria mellea and Trichoderma atroviride (cluster 1). Significantly enriched GO terms (P < 0.001) were identified using the BiNGO tool [42] and visualised with Cytoscape software [43]. The colour scale legend indicates the level of significance for enriched GO terms. White nodes indicate not significantly overrepresented categories. (PDF 111 kb) [file 12864_2016_3174_MOESM11_ESM.pdf]

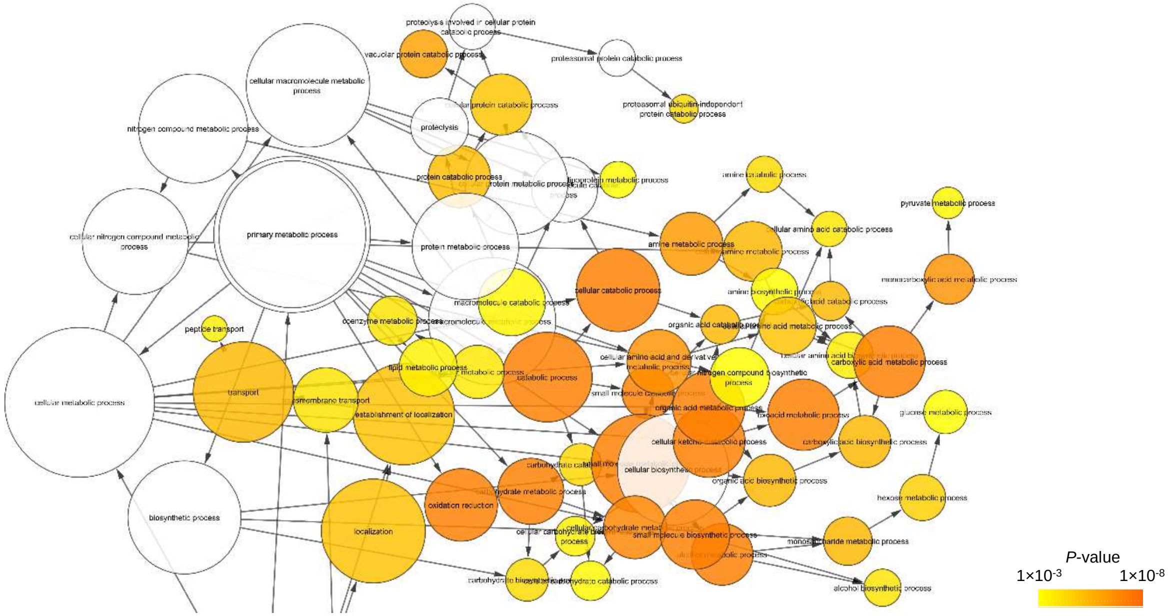

Additional file 11. Biological networks of Gene Ontology (GO) terms. GO biological process terms of the simplified soil microcosm, upregulated by incubation in the soil matrix with similar expression profiles in the presence or absence of *Armillaria mellea* and *Trichoderma atroviride* (cluster 1). Significantly enriched GO terms ($P < 0.001$) were identified using the BiNGO tool [39] and visualised with Cytoscape software [40]. The colour scale legend indicates the level of significance for enriched GO terms. White nodes indicate not significantly overrepresented categories.
